# Designing Flexible Longitudinal Regimens: Supporting Clinician Planning for Discontinuation of Psychiatric Drugs

**DOI:** 10.1145/3491102.3502206

**Published:** 2022-04-29

**Authors:** Eunkyung Jo, Myeonghan Ryu, Georgia Kenderova, Samuel So, Bryan Shapiro, Alexandra Papoutsaki, Daniel A. Epstein

**Affiliations:** University of California, Irvine, USA; Independent Researcher Irvine, USA; University of Washington, USA; University of Washington, USA; University of California Irvine, Medical Center, USA; Pomona College, USA; University of California, Irvine, USA

**Keywords:** Clinical decision support systems, Planning, Psychiatric drugs, Antidepressants

## Abstract

Clinical decision support tools have typically focused on one-time support for diagnosis or prognosis, but have the ability to support providers in longitudinal planning of patient care regimens amidst infrastructural challenges. We explore an opportunity for technology support for discontinuing antidepressants, where clinical guidelines increasingly recommend gradual discontinuation over abruptly stopping to avoid withdrawal symptoms, but providers have varying levels of experience and diverse strategies for supporting patients through discontinuation. We conducted two studies with 12 providers, identifying providers’ needs in developing discontinuation plans and deriving design guidelines. We then iteratively designed and implemented AT Planner, instantiating the guidelines by projecting taper schedules and providing flexibility for adjustment. Provider feedback on AT Planner highlighted that discontinuation plans required balancing interpersonal and infrastructural constraints and surfaced the need for different technological support based on clinical experience. We discuss the benefits and challenges of incorporating flexibility and advice into clinical planning tools.

## 1 INTRODUCTION

Growing adoption of electronic medical records (EMRs) has led to broader interest and adoption of clinical decision support tools, which are technology designed to support medical decision-making [59]. Clinical decision tools primarily focus on one-time decision support, such as diagnosis of a disease [8, 13, 14], patient assessment [49, 50], and prognosis predictions [77, 78]. However, care for many medical conditions, including irritable bowel syndrome [16, 39, 69], infertility [21, 22], and depression [34, 55], requires longitudinal planning involving multiple clinical decisions during treatment. Designing clinical decision support tools for longitudinal planning is challenging, as it requires continuous monitoring and assessment of patient conditions and frequent adaptations of treatment plans [38, 78]. The sociotechnical contexts of planning and delivering healthcare involving multiple stakeholders, such as different types of providers, pharmacies, and insurance companies, make designing clinical tools for longitudinal planning support even more complex [62, 64, 73]. Research has shown technology’s benefits in assisting longitudinal planning for health in non-clinical settings, such as providing informational support to help people generate behavioral plans and offloading the burden of articulating and scheduling small steps to reach their health goals [4, 48, 68]. However, we have a limited understanding of how to design technology to support the informational and logistical needs of longitudinal planning around clinical conditions while accounting for the human infrastructure [46] from a sociotechnical perspective [9].

We examine clinical decision support technology for longitudinal planning of discontinuation of antidepressants. Since the discontinuation of antidepressants often involves debilitating withdrawal symptoms which can last for months or even years [18, 66], prevailing expert opinion recommends gradual discontinuation over months (a *taper*) rather than abrupt cessation, while carefully monitoring symptoms [33]. Tapering antidepressants often requires iterative revision of the schedule to adapt to the patient’s reactions [33] and may involve non-standard prescriptions to achieve a sufficiently gradual taper [33, 35, 52, 71]. However, these revisions require buy-in from stakeholders such as pharmacies and insurance companies [32, 70]. Further, there is a lack of clinical research and evidence-based guidelines on how to gradually discontinue antidepressants to mitigate withdrawal symptoms [33, 58, 60]. Therefore, providers devise protocols based on their clinical intuition and often struggle if lacking experience in tapering antidepressants. Tapering antidepressants is, therefore, a useful case study for understanding how technology can support providers in developing longitudinal care plans amidst infrastructural challenges.

To understand how technology can better support longitudinal clinician planning, we iteratively designed and evaluated a clinical decision support tool for assisting providers in developing longitudinal plans for tapering antidepressants. Before designing the tool, we conducted two formative studies with eight providers with varying levels of experience and who regularly prescribe antidepressants. We first understood their strategies for tapering antidepressants and how technology can and should support their practices, verifying this understanding with a low-fidelity prototype based on design guidelines we developed. After refining these guidelines, we designed and implemented a high-fidelity prototype, AT Planner. AT Planner scaffolds the taper planning process by supporting flexible prescription configuration, projects a full prescription plan which providers can adjust to fit their clinical regimens or align with patient needs, and then automatically creates notes for sharing with pharmacies and patients based on selected prescriptions.

Through a feedback study on AT Planner with eight providers, we found that providers’ taper planning practices were under interpersonal and infrastructural constraints, facing barriers from pharmacies and insurance companies in creating the complex prescriptions required for longitudinal plans. We also found that providers desired different types of technology support based on their varying levels of experience. Providers with more experience in tapering antidepressants, typically psychiatrists, preferred that technology supports greater flexibility in planning to allow them to adapt taper schedules to their current practice and react to patients’ experiences. Conversely, providers with less experience in tapering antidepressants, such as general practitioners, often wished technology could automate the process of creating the taper plans, such as suggesting and generating standard taper schedules. Based on the findings, we examine how technology can better support providers in balancing the influencing constraints on developing care plans, particularly around longitudinal planning. We also suggest that providers’ varying levels of experience need to be carefully considered in the design of clinical decision support tools. Lastly, we consider the opportunities and challenges of clinical decision support tools operating outside the EMR systems in research and in practice.

The key contributions of this work include:

- Design guidelines for clinical decision support tools that assist longitudinal planning, emphasizing the needs for flexibly supporting providers’ various regimens, scaffolding longitudinal decision-making through iterative planning, and seamlessly integrating into clinical workflows.
- Design and implementation of AT Planner, which scaffolds longitudinal taper planning by projecting schedules, allowing flexible adjustment, and generating notes to connect to the EMRs. Providers’ feedback on AT Planner pointed to influences of interpersonal and infrastructural constraints, resulting in taper plans which balanced conflicting needs and desires for different types of technology support based on their clinical experience.
- Implications for research on clinical decision support tools, particularly around 1) providing greater flexibility, even allowing some loopholes to help providers balance interpersonal and infrastructural constraints, 2) understanding the experience levels and needs of the target providers before designing tools, 3) understanding the benefits and challenges of developing tools operating outside the EMRs in enabling prototyping and evaluating design ideas versus limitations when extending to clinical adoption.

## 2 BACKGROUND: PRESCRIBING AND DISCONTINUING ANTIDEPRESSANTS

The rising prevalence of diagnoses of mental health disorders in the United States [3] has coincided with a marked increase in the prescription of psychiatric drugs. One in six adults in the U.S. report filling one or more psychiatric drug prescriptions annually, with antidepressants being the most commonly prescribed class of psychiatric drugs [6, 63]. Clinical guidelines increasingly suggest considering discontinuation of antidepressants when patients achieve complete symptom remission for a prolonged time. The U.K. National Institute for Health and Care Excellence suggests supporting discontinuation of an antidepressant if a patient has been on it for more than six months after remission of an episode of depression [23]. The American Psychiatric Association also states that antidepressant treatments can be discontinued for stable patients, though the precise timing has not been convincingly determined [7]. Even before achieving symptom remission, discontinuation of antidepressants is considered when patients experience significant side effects [67], perceive ineffectiveness of the medication [12], or have other conditions (e.g., pregnancy) that may be adversely impacted by ongoing antidepressant treatment [74].

Discontinuing antidepressants is a challenging task. A systematic review article reported that more than half of the people who attempt to come off antidepressants experience withdrawal symptoms which may include flu-like symptoms, insomnia, nausea, or sensory disturbances [18, 66, 67, 74]. Withdrawal symptoms have been frequently reported with SSRI (Selective-Serotonin Reuptake Inhibitor) and SNRI (Serotonin-Norepinephrine Reuptake Inhibitor) antidepressants [67]^1^. Antidepressants with shorter half-lives (i.e., the length of time required for a drug to decrease to half of its starting dose in the body [31]) are more likely to evoke withdrawal symptoms with greater severity compared to antidepressants with longer half-lives [33, 66]. These withdrawal symptoms can be debilitating and last for months and even years [18, 66], and can lead to serious psychiatric problems such as suicidal ideation [30].

Recent research has provided theoretical evidence that exponential taper of antidepressants (i.e., making dose changes based on fixed percentage reductions in dose such that tapers become more gradual towards the end) over months, as opposed to linear taper (i.e., making fixed numerical dosage amount (e.g., milligram) reductions) might help prevent withdrawal symptoms [33]. Clinical recommendations advise gradual discontinuation (a taper) rather than abrupt cessation [7, 23, 65]. Since tolerance for dose reductions could vary by individual, studies suggest that tapering plans should be constantly tailored by carefully monitoring individual patient reactions [33, 67]. For example, providers may adjust the reduction rate, switch to another drug form (e.g., from tablet to liquid), or a different kind of drug. Switching to a different drug is called *“cross-taper”* [41], which typically means switching from an antidepressant with a shorter half-life to another antidepressant with a longer half-life to mitigate withdrawal symptoms.

Unfortunately, there is no standard approach on how to plan for the gradual discontinuation of antidepressants [33, 58]. Instead, current recommendations are vague, suggesting tapers *“over the course of at least several weeks”* [7] or *“at a rate proportional to the duration of treatment.”* [23] Therefore, providers must independently devise taper regimens for individual patients based on their training and experiences. In the U.S., antidepressants are widely prescribed by primary care providers, including general practitioners and nurse practitioners, as well as psychiatric providers [76]. While primary care has benefits in terms of consistency, continuity, and accessibility [37, 72], the training that primary care practitioners receive on antidepressant treatment can be highly variable [76]. As a result, general practitioners may feel less confident about administering tapering regimens compared to psychiatrists [41, 58]

In addition, tapering antidepressants involves complex pharmacological considerations and may require incorporating different dosage formulations to achieve a sufficiently gradual taper. Antidepressants can be available for prescription as tablets, capsules, or liquid formulations. A recent survey with general practitioners and psychiatrists demonstrated that both providers predominantly used tablets or capsules (93-96%) whereas using liquid form was relatively uncommon (19-21%) [52]. Tablets are preferred by providers for both general-purpose and tapering prescriptions as they are more economical for patients and help facilitate flexible dose changes, especially when scored (i.e., embossed with a line to facilitate splitting in half) [35]. However, the available tablet dosage strengths of antidepressants are generally too high to allow for a significant gradual taper and several antidepressants are available only in the form of capsules or unscored tablets [71]. Liquid formulations allow even greater flexibility for creating smaller or intermediate doses to facilitate a gradual taper [33, 42] and can be an alternative for people who have difficulty swallowing pills [2]. However, they tend to be costly [71] and measuring accurate dosages can be challenging [2, 71]. In addition, insurance companies may reject or require prior authorization for non-standard formulations of antidepressants such as liquid [32, 70].

## 3 RELATED WORK

Supporting providers’ longitudinal planning for discontinuation of psychiatric drugs draws on past HCI work on technology support for clinical decision-making, planning for health, and sociotechnical aspects of healthcare.

### 3.1 Technology Support for Clinical Decision Making

Clinical decision support tools have increasingly been proposed as promising ways to assist providers with computational support in various medical domains such as detection or diagnosis of a disease [8, 13, 14, 29], patient assessment [49, 50], and prognosis prediction [77, 78]. Prior clinical decision support tools have leveraged automation to reduce human errors and mitigate the cognitive burden on providers [25, 51, 54]. Although previous studies have shown potential benefits of technology in assisting clinical decision-making, those systems have rarely been adopted in clinical practice [20, 36, 40, 57]. A frequently-mentioned barrier is that decision support tools are often a poor contextual fit in clinical workflows [40, 54, 75]. For better adoption of clinical decision support tools into clinical practice, studies have highlighted that such tools should be integrated into the existing healthcare systems such as EMRs [34, 38, 77, 78].

Additionally, providers are not likely to adopt clinical decision support technology if they feel it undermines or infringes their expertise [43, 73, 78]. Therefore, researchers increasingly highlight the need for reconsidering the relationship between the agency of providers and automation in the design of clinical decision support tools [13, 44, 51, 73, 77, 78]. Wang et al. [73] proposed framing clinical decision support tools as *“doctor assistants”* rather than replacements or replications of doctors, emphasizing the need for a clear division between what tasks can be automated and what tasks should be administered by providers. Studies have described different types of tasks that providers and machines can perform well, respectively. While computational support can be of great help with numeric-based tasks that generate objective output, human attention is required for tasks that need initiative and creativity [51]. In addition, while data-driven technology can help clarify and monitor patient conditions, clinical intuition is necessary when the decision requires balancing various clinical evidence and complex social evaluations [78]. Recent systems have leveraged AI for decision-making and interpretation, showing that giving providers agency to collaborate with technology can improve acceptance and effectiveness of clinical decision support tools [13, 49].

### 3.2 Technology Support for Planning and Scheduling for Health

Prior clinical decision support tools have predominantly focused on supporting making a single decision at a time [78]. However, care for many medical conditions, such as irritable bowel syndrome [16, 39, 69], infertility [21, 22], and depression [34, 55], often requires longitudinal planning and multiple decisions over time. HCI researchers increasingly investigate the need for technology to support longitudinal clinical decisions [29, 38, 78]. For example, Yang et al. [78] found that a heart implant decision requires many smaller clinical decisions which clarify, adapt, and optimize based on evolving patient conditions. Similarly, through their field study with clinical tools for assisting volume therapy decisions in intensive care units, Kaltenhauser et al. [38] suggested that clinical support tools should support decisions over time rather than providing a conclusive diagnosis or prognosis. Previous studies have pointed to the challenges of longitudinal care planning, as it requires continuous monitoring and assessment of patient conditions and adapting treatment plans accordingly. Technology has an opportunity to support longitudinal care planning by providing informational and logistical support to help providers adapt care plans.

Outside clinical settings, research has investigated technology’s role in assisting planning for managing and improving diverse domains in health, such as exercise [4, 5], diet [5], sleep [47], and mental health [48, 68]. Technology can provide substantial informational support to help people generate effective plans. For example, by providing expert guidelines and a database of physical activities, Agapie et al. [4] helped crowdworkers plan out the amounts and what kind of health activities to do. Planning support systems such as MUBS [68] have also provided personalized recommendations for activities to do. In addition, technology can help people develop plans which provide answers to specific questions that people might have, such as TummyTrials [39] helping people plan a scientific process to determine what causes their symptoms and SleepCoacher [17] helping determine what causes their symptoms or health outcomes.

Creating effective long-term plans for health requires articulating and scheduling small steps to reach the goal. For example, when creating exercise plans, one needs to identify and articulate what activities to perform, how much activity to perform, and when to perform activities each time [4]. Technology can provide logistical support to help long-term planning by offloading the burden of articulating and scheduling small steps to reach individuals’ health goals. For example, MindForecaster [48] and MUBS [68] supported scheduling time to work on health activities, such as intervention plans to cope with anticipated stressful situations or activities to promote mental health. Studies have suggested that humans’ insights are vital for balancing complex individual preferences, constraints, and expert guidelines for personalizing plans [4]. Research further highlights the importance of making adaptations in light of individuals’ evolving health status, knowledge, and contexts. For example, CrowdFit [4] let planners adjust exercise plans based on the clients’ feedback, and Lee et al.’s system [47] allowed individuals to modify what behavior change technique their plan leveraged.

### 3.3 Sociotechnical Aspects of Providing Healthcare

Human infrastructure, or the human and organizational arrangements that are required in order for collaborative work to be accomplished [46], is critical for understanding clinical care because most of the modern healthcare service is planned and delivered through collaborative efforts among different types of stakeholders. Berg’s sociotechnical approach characterizes healthcare work as ongoing negotiations among various professionals with different viewpoints and potentially conflicting goals [9]. This approach highlights the need for considering the interdependence among different professionals when designing clinical tools [9, 10].

Researchers in medical informatics have argued the need for considering the collaborative nature of clinical work when designing computerized physician order entry (CPOE) systems [1, 27, 57], which are systems physicians use to send prescriptions to pharmacies. Most CPOE systems focus on supporting tasks of individual physicians [1, 27]. However, medical orders are created and processed by complex interactions of physicians, nurses, pharmacists, and other types of health professionals. Ignoring the collaborative aspects of medical orders could thus lead to interruptions in the clinical workflow [1, 57]. For example, in a paper-based prescribing environment, pharmacists traditionally played a role in verifying and making low-risk amendments of prescription orders by annotating the prescription sheet. However, pharmacists became no longer able to continue the fine-tuning practice with the CPOE systems [64], which resulted in an increase in communication load for both pharmacists and physicians for verifying the orders [64]. Similarly, Patterson et al. [62] showed that relative to paper-based free-text notes, the introduction of a CPOE system made it inefficient for pharmacists to process non-standard prescriptions such as tapering medication doses.

Insurance policies and pharmaceutical companies also influence the planning and delivery of healthcare. Wang et al. [73] described how Chinese insurance policies, such as rejecting reimbursements for cases that were deemed as inappropriate (e.g., overuse of antibiotics), impacted providers’ prescription practices. Groot et al. [28] illustrated another example where many health insurers in the Netherlands refused to reimburse tapering antidepressants to patients with severe withdrawal symptoms whom their providers wanted to administer gradual tapering schedules over longer periods of time. Recent studies criticized the lack of dose pill options provided by pharmaceutical companies, as it can lead to frequent prescriptions of tablet-splitting despite the potential risk of dose inaccuracy [35, 71].

To sum up, healthcare providers engage in ongoing negotiations with multiple stakeholders and technology, balancing conflicting views and influences to provide care to their patients. In our work, we grow our understanding of how to design clinical decision support tools to support longitudinal planning, balancing the needs of the stakeholders.

## 4 METHODS

**Figure 1:**
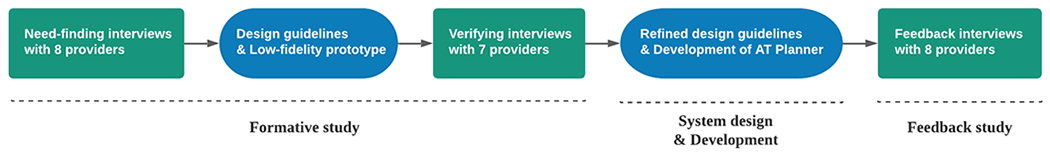
We designed and evaluated AT Planner via a formative study, system design & development, and a feedback study.

To understand providers’ perspectives on the role of clinician tools for antidepressant taper planning, we conducted two studies: a formative study and a feedback study. The formative study aimed to understand the current practices of providers when tapering antidepressants and how technology can support their needs. The formative study consisted of two rounds of interviews with eight providers who have prior experience supporting patients in tapering antidepressants but come from different clinical backgrounds. We conducted the first round of interviews for need-finding, developing a low-fidelity prototype based on the insights, and the second round of interviews for verification of providers’ design needs, with seven out of eight providers returning. Based on the study findings, we developed design guidelines for a clinical support tool for planning antidepressant tapers, which guided our implementation of AT Planner. We then conducted a feedback study with AT Planner, consisting of interviews with eight providers. The feedback study examined the potential impact of technology that supports flexible planning for discontinuing antidepressants in clinical practice, using AT Planner to elicit providers’ thoughts. We interviewed 12 different providers in total, with four participating in both studies. The study procedures are summarized in Figure 1.

### 4.1 Study Procedures

#### 4.1.1 Formative Study.

The formative study consisted of two interviews: the first round of interviews for need-finding and the second round of interviews to verify our interpretations of providers’ design needs. Due to the COVID-19 outbreak, all research activities were conducted remotely using video conferencing via Zoom. Two members of the research team conducted each interview, with one asking protocol questions and the other asking probing questions.

In the first round of interviews, we sought to understand providers’ perspectives on tapering antidepressants and how technology can and should support their practices. We first understood their current practices tapering a patient off of an SSRI/SNRI antidepressant by asking them to imagine developing a taper for a patient based on their dosage and mental health condition. We then sought to better understand their approaches to managing tapering antidepressants, including how they calculate taper dosages and how they adjust plans when patients experience withdrawal symptoms or relapses of depressive symptoms. Finally, we asked them how technology could improve the tapering management process, such as what kind of features they would like a taper planning tool to have and in what contexts they would like to use such a tool. Based on findings from these interviews, we developed five preliminary design guidelines for supporting planning for tapering antidepressants. Following these guidelines, we developed a low-fidelity prototype of a tool to support tapering using Mockflow.

In the second round of interviews, we sought to verify our interpretation of providers’ design needs with the low-fidelity prototype. We walked participants through the prototype and asked questions to elicit feedback, such as whether the features would be helpful for configuring tapers in their practice and if there would be any additional features that might be useful. After the interviews, we revised our design guidelines into four guidelines. Section 5 describes our refined guidelines. The supplemental material contains our formative study interview protocols and screenshots of the low-fidelity prototype.

#### 4.1.2. System Design and Development.

We then developed a high-fidelity prototype, AT Planner, which is a realized version of our design guidelines derived from our formative study findings. We walk through the full system design in Section 6. We implemented AT Planner using React in TypeScript. We chose to develop a web application to enable providers to participate in our feedback study from home or in their office by running it on their machines without having to install dedicated software. Because we only implemented the client-side, participants’ input and output data (e.g., participant-generated projected schedules) only persisted in the browser session and was not stored. The application is publicly accessible at: https://pielab-uci.github.io/antidepressant-tapering/.

#### 4.1.3 Feedback Study.

We conducted a feedback study with eight providers, using AT Planner to scaffold broader conversations around the utility of the tool’s concepts for supporting providers in tapering antidepressants and the benefits and challenges of integrating those concepts into clinical practice. The feedback study involved a 60-minute Zoom meeting with each participant, facilitated by one or two members of the research team. During the study, we sent providers the link to AT Planner and asked them to share their screen and think aloud as they interacted with the tool. Our study used AT Planner as a backdrop for understanding the role of technology in the space of planning tapers, rather than evaluating providers’ ability to use AT Planner to complete predefined tasks or the usability of the specific interfaces. Therefore, we provided guidance to the providers when they found an aspect of the interface confusing or unintuitive.

We first explained the overall concept of AT Planner, focusing on the iterative planning aspect. Next, we asked participants to come up with an example patient who is planning to be entirely off of an antidepressant and use AT Planner to develop the tapering plan. We then asked providers to imagine that they found the patient struggling with withdrawal symptoms in their follow-up visit, and to make adjustments to the tapering plan accordingly. Once participants had a sense of the capabilities and structure of AT Planner, the interview broadened to ask providers about the potential impact of technology like AT Planner on their practice. The interview questions asked how they felt about using technology to create and adjust a tapering plan compared to their current approach and which of the typical care plans they administer would be well- or poorly supported through AT Planner or technology more broadly.

During the feedback study, we regularly met as a full research team and reflected on the participants’ feedback to better fit into their clinical practice. We iteratively refined some aspects of the tool design after each interview, in addition to fixing small usability and performance bugs. For example, the default projection mode of AT Planner was exponential, but we added a feature allowing providers to switch to linear per participants’ feedback. We also made iterative wording and format changes to patient instructions and notes for pharmacies based on participants’ feedback.

#### 4.1.4 Data Analysis.

All interviews were video-recorded, automatically transcribed through Zoom, and manually revised to correct errors afterward. We used thematic analysis [11] to qualitatively analyze both interview studies. The first author open-coded the transcripts to identify patterns in the dataset. The full research team discussed and identified themes. The final codebook contained nine parent codes and 32 child codes for the formative study and seven parent codes and 22 child codes for the feedback study. One of our co-authors, a licensed psychiatrist, regularly reviewed our findings to verify if the conclusions from our analysis aligned or conflicted with medical expertise.

### 4.2 Participants

We recruited providers through mailing lists associated with the Psychiatry Department at our University’s Medical Center, other affiliated psychiatric care sites, and through direct recommendations of a psychiatric provider in our research team. We required providers to have prior experience supporting patients in tapering SSRI or SNRI antidepressants. Both formative and feedback studies were classified as exempt by our University’s Institutional Review Board because the interview methodology did not involve more than minimal risk to participants, and any disclosure of participants’ responses would not place participants at the risk of damaging their employability or reputation.

Three psychiatrists, three nurse practitioners, and two general practitioners participated in the formative study (Table 1). All eight providers participated in the first interview, with all returning for the second interview except GP2. We compensated each participant $25 cash or a gift card for two 20-30 minute individual interview sessions. Four practicing psychiatrists, one psychiatric resident, and three general practitioners participated in the feedback study (Table 1). We compensated each participant $50 cash or a gift card for a one-hour individual interview session. Most participants’ primary affiliation was either the Family Medicine or Psychiatry Departments of our University Medical Center. All nurse practitioners were affiliated with private practices, and GP4 was affiliated with a Community Mental Health Center. Participants varied in clinical experience, from last year of residency or a few years post-residency to a decade or more of practice.

### 4.3 Limitations

We sought to understand provider perspectives on the main design components of AT Planner, such as scaffolding longitudinal planning and enabling flexible adjustments. We expected that provider burdens of using a tool operating outside of EMR settings in clinical environments would make it challenging to get feedback on the design components, which we discuss in detail in Section 8.3. We thus decided to focus on gaining feedback on prototypes through interview studies without imposing that burden on providers rather than to conduct a field deployment of AT Planner. Further longitudinal evaluation of AT Planner in clinical environments, particularly around integrating into EMR systems, is likely to surface additional challenges in designing clinical tools and further contribute to our understanding of how to support longitudinal planning.

Past work has highlighted that patients are often self-motivated to discontinue psychiatric drugs, with or without the support of a provider [24, 28, 60]. We focused on investigating clinical support for taper planning because the clinical recommendation to support tapering is increasing, but guidance is currently low, which indicates a need to understand how to support providers in developing tapers. Engaging patients in the design process is likely to reveal additional needs for clinician taper planning to account for their perspectives. For example, documenting patients’ withdrawal symptoms can benefit both patients and providers as it allows providers to adjust their prescriptions based on patients’ felt experiences, particularly when structured for easy review [60]. Having a deeper understanding and incorporating patient needs into the design of taper support tools is an important area of future research.

All of our participants except GP4 were working for either a University Medical Center or in private practice in a relatively wealthy city in the United States. Our participants described their practices in prescribing and discontinuing antidepressants as being influenced by the socioeconomic statuses of patients. For example, patients in Community Mental Health Centers tend to have less frequent consultation times with their providers compared to University Medical Centers or private clinics. Providers’ experience in this specific domain, providers’ relationship with pharmacies and insurance companies, what EMR systems are being used in providers’ medical systems, and how healthcare systems are designed also impact their clinical practices around discontinuation of antidepressants. Therefore, our findings might not generalize as well to different medical settings in different geographical locations in the United States or other countries with healthcare systems designed in a different way.

## 5 FORMATIVE STUDY FINDINGS: DESIGN GUIDELINES

Based on the insights that we gained through the formative study with eight providers, we developed the following design guidelines that clinical decision support tools for tapering antidepressants should follow.

### 5.1 Flexibly Supporting Providers’ Various Regimens

Participant’s perspectives suggest that taper planning tools should allow for a range of different tapering regimens. Previous work has pointed out that there are no specific clinical guidelines for how providers should support tapering off antidepressants to minimize withdrawal symptoms [33, 58]. Our participants acknowledged the lack of clinical guidelines and described how they devised their own strategies for tapering off antidepressants using their clinical intuition. All of them agreed with the need for gradual discontinuation of antidepressants as abrupt discontinuation can provoke withdrawal symptoms or reemergence of depressive symptoms. For example, NP2 said, *“I am really on the conservative side, so we would usually taper over four-week intervals (i.e., the duration of time on a certain dosage strength of a drug before reducing to a lower dosage), between 25% to 50% rate.”* However, we noted significant variability in each provider’s typical regimens. In terms of the reduction rate, two participants (GP2, PS3) said they would reduce antidepressants by 25% every interval, two participants (NP2, NP3) said they would reduce them by 25% to 50% every interval, and three participants (NP1, PS2, GP1) said they would reduce them by 50% every interval. Providers’ regimens varied in interval length, as well, from frequent reductions every one-two weeks (GP1) and every two weeks (GP2, NP1) to longer or more varied intervals like four to eight weeks (PS3) or every couple of months (PS2). In addition, providers often desired gradually introducing another antidepressant while decreasing the other simultaneously (cross-taper), as well as tapering off a single antidepressant. They hoped that decision support tools could also assist cross-tapering.

Even though the providers usually applied their own rules in terms of certain reduction rates and intervals, they still noted tapers should be *“customized to the patient”* (PS1) considering various factors specific to each patient. The factors that the providers considered included symptom history (e.g., how long the patient has experienced depression), medication history (e.g., how long the patient has been on the medication), history of the patient’s reaction to the medication (e.g., whether they have experienced withdrawal symptoms in their past tapering attempts), and current dosage (e.g., whether it is the maximum available one). For example, if a patient has experienced multiple episodes of depression and been on antidepressants for a few years, some prescribers described implementing a more gradual taper than if they were tapering a patient off of medication after a single episode that lasted a few months. Similarly, if patients had unsuccessful attempts to discontinue their antidepressants previously, providers wanted to take a more careful approach. Providers also aimed to respect patients’ willingness and feelings towards the tapering speed and potential withdrawal symptoms. When patients felt confident and comfortable about tapering at a faster rate (e.g., 25% every two weeks rather than every four weeks), providers often supported them in doing so. PS3 noted, *“I will probably follow the guide of the patient. If they feel they can tolerate this, sometimes I may give them a short-term one.”*

In addition, when providers wanted to incorporate smaller and intermediary doses that they could not directly order from a pharmacy into the taper, they used different strategies. Providers mentioned using compound pharmacies and liquid formulations. However, there were some challenges associated with these methods, such as insurance coverage and pharmacy availability. Therefore, the most common and standard strategies to obtain smaller dosages include using pill cutters to cut scored tablets in half. NP3 preferred using a pill cutter to get smaller dosages as it is a simpler method than others: *“Compound pharmacy or liquid is not always available or covered by insurance, so they are kind of down the list. So I would either use the next dosage available or cut the pills in half to decrease the dosage.”* Providers (GP1, PS1, PS3) also wanted to make use of patients’ leftover pills for getting smaller dosages by using a pill cutter. GP1 said, *“If you have extra 100 (mg pills), and the next step I wanted to do was to go down to 50 (mg), you can just cut it in half, instead of having to buy more.”*

Considering the complex needs in the tapering process, taper planning tools need to support flexibility in terms of reduction rate, intervals, cross-taper, and different methods for micro-dosing.

### 5.2 Seamlessly Integrating into Clinical Workflows

Providers repeatedly emphasized the difficulties of integrating taper planning into their workflows, suggesting that planning needed to be quick and easy as their typical follow-up appointments are short (e.g., from three minutes to twenty minutes). GP1 said, *“being able to do it fast is really important. If you have a 20-minute appointment and that (taper planning) really ends up being like ten minutes, then you don’t have a lot of time to do face-to-face time with patients.”* We found that providers often had to enter the same information when prescribing medications to multiple platforms such as EMRs and after-visit notes for patients. Therefore, they were concerned that introducing a new tool to clinical settings might create even greater redundancy. PS1 noted, *“Doing a taper, residents would write it down on the prescription sheet and then put it in the after-visit summary which should be given to the patient. And then if they are going to program it into an app, that’s a lot of the exact same data, so that might be annoying.”* To mitigate the burden from double-charting, they hoped that the tool would generate prescription information that could be easily transferred to their existing platforms. PS2 imagined, *“If it calculates the taper for you and you can just copy and paste it into the after-visit summary, it would be nice and easy.”* Similarly, PS1 stated, *“If it generates the [prescription] text on its own and it’s easy enough to copy-paste into the order for prescription, that’d be really convenient.”*

### 5.3 Articulating Longer-Term Plans

Participants wanted taper tools to describe the full taper plan, preferring to plan out all dosage changes at once. GP1 preferred planning the taper out on the first day as his typical patients did not come back frequently. Therefore, he usually gave patients instructions involving a couple of steps of dosage reductions: *“I would tell them to cut it in half for two weeks, then cut it in half again for two weeks. If you have any problems, you can always come back. If not, you’ll be back in a month, and hopefully, by then, you’ll be completely off of it.”* Since the tapering schedules might involve multiple steps of dosage change, providers highlighted the need for considering ways to better communicate the complexity of the tapering schedule. NP2 said he would often experience challenges in communicating the taper schedules with patients. He described, *“We try to write it [taper schedule], and they get a medication label from the pharmacy, but still, miscommunication does happen. Anything that would make it easier for patients to comply with the taper schedule would be beneficial.”* NP1 empathized with this need and hoped to have a clearer way to communicate the multiple steps of prescriptions to patients during the taper as well: *“Our notes are clear to us; we know what we’re thinking. But that can sometimes get misconstrued to patients. […] I do find tapering sometimes messed up because patients are just getting confused when to increase or when to decrease.”*

### 5.4 Configurable through an Iterative Process

Providers suggested that the taper process needed to be adjustable. While some of the participants preferred to plan out the whole taper, including multiple dose changes all at once, they often iteratively revised the taper plans in light of patients’ reactions to dose changes. For instance, GP2 explained her tapering strategy as to *“move to the next dose down in the follow-up every two weeks.”* This practice ensures that they can prevent and manage withdrawal symptoms and relapse of depressive symptoms. A follow-up visit was an important space for providers to determine whether they should stick with or adjust tapering schedules based on patients’ tolerance of the previous dosage change. If patients reported at their follow-up visits that they had experienced a relapse of depressive or withdrawal symptoms, providers adjusted the tapering schedules to mitigate the symptoms. NP3 described the tapering process as *“reducing the dosage and then having follow-up visits with patients to determine the response.”* Their standard practice for adjusting the taper was to *“go back to the previous dose that symptoms were controlled”* (PS1, NP2, GP1) and *“slower the taper down”* (GP1, PS3, GP2) by increasing the taper rate and/or increasing the length of intervals. PS3 would thus emphasize to his patients that the tapering schedule of antidepressants is tentative and subject to change: *“It doesn’t have to be hard and fast. It’s not like an antibiotic that you really have to finish the course even if they’re feeling better. We convey that there may need to be potential adjustments.”*

## 6 SYSTEM DESIGN

Informed by our formative interviews, we designed and developed AT Planner, a web application for psychiatric providers to help them iteratively develop plans for tapering antidepressants while accommodating their care regimens.

### 6.1 System Overview

AT Planner scaffolds the process of planning antidepressant tapers by allowing providers to select from different options of available dosages for each drug and formulation. Based on the selected projection mode and the difference between the current and next dosage, AT Planner populates a tentative taper schedule projecting future prescriptions until the patient reaches the goal dosage. Then, AT Planner allows providers to flexibly adjust the taper plans to customize to their regimens. We envision that providers could use AT Planner to revisit the projected schedule in the patient’s follow-up consultations, making iterative adjustments in light of patients’ reactions to the taper. Once providers select what intervals they would like to prescribe, the system automatically creates notes for pharmacies and patients in plain text to be easily shared through existing healthcare systems.

### 6.2 System Features

Informed by our design guidelines, AT Planner has four main features: scaffolding taper planning, projecting tentative schedules, and providing flexibility to adjust taper plans.

#### 6.2.1 Scaffolding Taper Planning.

**Figure 2:**
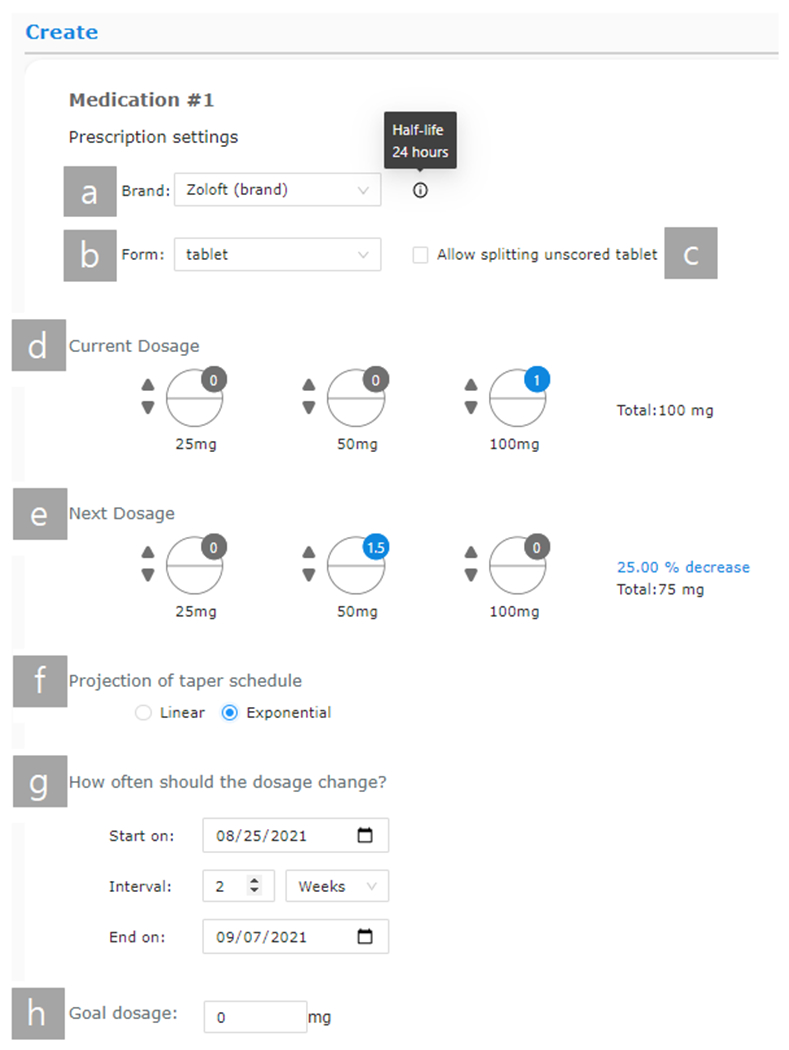
To configure a taper plan in AT Planner, providers choose (a) the medication brand, (b) an available drug form, and (c) whether splitting unscored tablets is allowed. Providers then indicate (d) the dose a patient is currently on, (e) the dose to be prescribed next, (f) what mode future projections should take, (g) the duration of each interval, and (h) the goal dosage for the end of the taper.

To scaffold taper planning, AT Planner provides information relevant to prescribing SSRIs and SNRIs. Providers are first given medication options of five of the most frequently prescribed SSRIs and SNRIs [56] in both brand-name and generic version: Prozac / Fluoxetine, Citalopram / Celexa, Sertraline / Zoloft, Paroxetine / Paxil, and Escitalopram / Lexapro. These medications were selected with the guidance of our psychiatrist co-author. AT Planner also provides information about the half-life of a chosen medication when hovering next to the medication options input (Figure 2 (a)). After selecting a type of medication (Figure 2 (a)), providers can choose from different formulations, either tablet, capsule, or liquid, depending on what options are available on the market for a prescription for each drug (Figure 2 (b)). Providers frequently look up information about medication’s available formulations, doses, and half-lives when developing tapers, so we retrieved them from GoodRx [26] and individual medications’ package inserts and incorporated them into AT Planner.

AT Planner also scaffolds taper planning by providing a visual interface to configure prescriptions. Once the brand and form of the medication are chosen, AT Planner shows registered dosage options for the corresponding formulation in the current dosage and the next dosage (Figure 2 (d, e)). The current dosage is the dose that a patient is currently on, and the next dosage is the dose that the provider intends to prescribe in the current consultation. For tablets and capsules, providers are given available dosage options to select how many of different strength pills to prescribe (e.g., 100 mg, 50 mg; Figure 2 (d, e)). The icons of pills change depending on whether they are capsules, scored tablets, or unscored tablets. By default, the count of capsules and unscored tablets change by one, but AT Planner allows prescribing half-doses for scored tablets. If providers feel their patients would be interested and capable of cutting unscored tablets, they can check *“Allow splitting unscored tablet”* (Figure 2 (c)). For liquid, when a dosage input is entered in mg, the input in ml is automatically calculated, and vice versa.

After setting the current and next dosage, AT Planner allows providers to choose from two options on how to project the taper schedule: linear or exponential (Figure 2 (f)). This input is used to populate the upcoming dosages of medication and create a tentative taper schedule. Depending on the chosen projection mode, projected dosages are calculated based on the difference between the current and the next dosage by rate (exponential) or amount (linear). The duration of each interval could be set by defining two of the start date, interval, and the end date (Figure 2 (g)). Lastly, providers could select a goal dosage to determine when the projection of schedules should stop, with the default being 0 mg (full discontinuation) (Figure 2 (h)). Providers can also develop cross-tapering plans. Once they add a new medication and follow the same steps, AT Planner generates projected schedules for both medications in parallel.

#### 6.2.2 Projecting Tentative Schedules.

**Figure 3:**
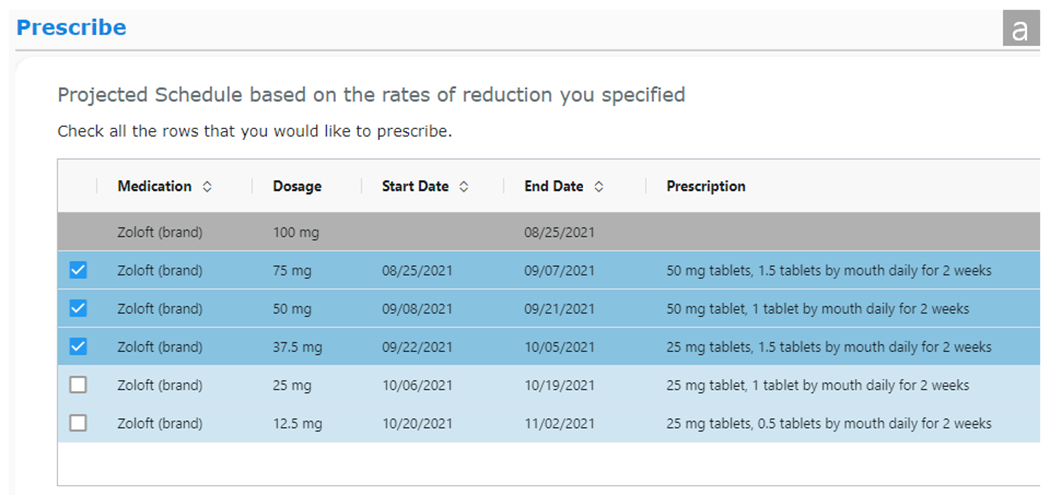

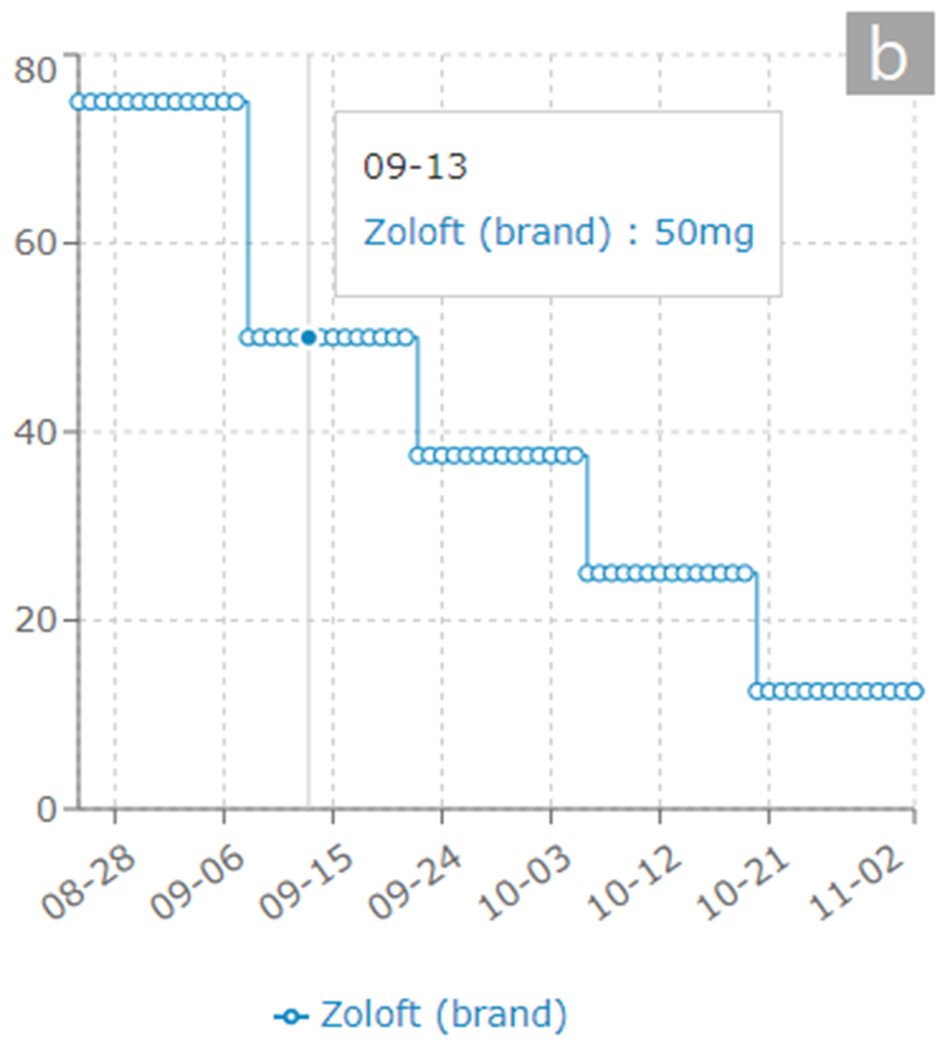
Based on the configured settings, AT Planner projects a potential taper schedule in a table and a line chart. (a) Each row in the table represents an interval, and selected are included in the notes for patient and pharmacy (see Figure 4b). (b) The line chart highlights the dosages and reduction rate across the schedule.

AT Planner enables providers to plan the entire course of a taper by projecting tentative schedules. Based on the entered dosage reduction projection type, interval duration, and goal, AT Planner generates a tentative taper schedule until reaching the goal dosage (Figure 3). AT Planner will approximate the reduction rate, opting for the closest lower available doses of tablets or capsules registered at pharmacies when exact dosages are not available. For example, if a provider developing a taper for Zoloft selected 100 mg for the current dosage and 75 mg for the next dosage, an exponential projection will project future dosages estimating a 25% reduction for each interval. The exponential projection would therefore project 75 mg, 50 mg, 37.5 mg, 25 mg, and 12.5 mg to approximate a 25% reduction with available dosages. Conversely, a linear projection would reduce by 25 mg each interval, projecting 75 mg, 50 mg, and 25 mg. For liquid, AT Planner will approximate the projected doses to the nearest whole number of milliliters (e.g., 5 ml). Each row in the table represents individual dose changes and corresponding intervals (Figure 3 (a)). In the prescription text, AT Planner suggests a combination of available dose options for the projected dose of each interval in a way that minimizes the total count of tablets or capsules. Let’s revisit the example above. Considering that Zoloft is available in 25 mg, 50 mg, and 100 mg scored tablets, AT Planner will suggest the combinations to obtain projected dosages as one and a half of 50 mg tablets (75 mg), one 50 mg tablet (50 mg), one and a half of 25 mg tablets (37.5 mg), one 25 mg tablet (25 mg), and a half of 25 mg tablets (12.5 mg). For liquid, AT Planner suggests how many and what size of bottles should be prescribed based on the total milliliters.

The projected dosages are also visualized in a line chart (Figure 3 (b)), allowing providers to see the overall plans at a glance. When providers select intervals that they would like to prescribe at a time, the selected rows are highlighted.

#### 6.2.3 Providing Flexibility to Adjust Taper Plans.

**Figure 4:**
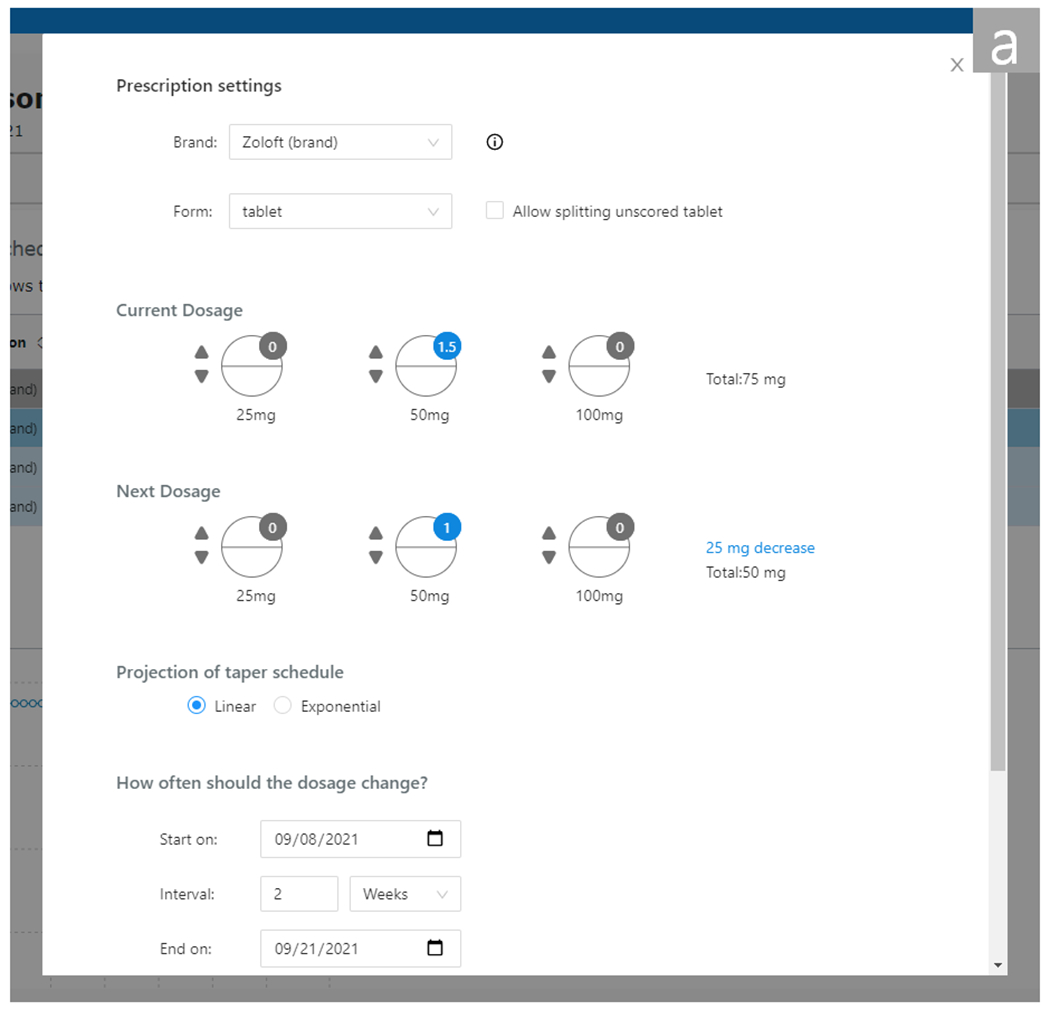

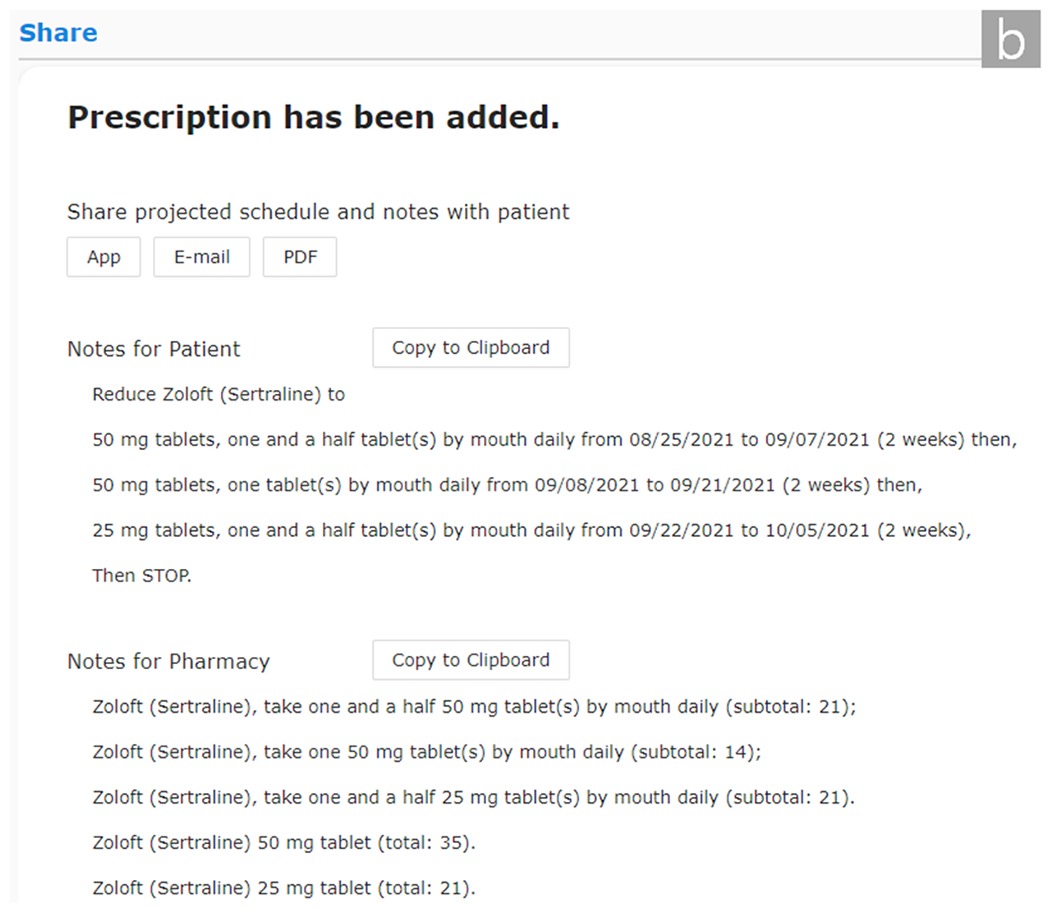
(a) Providers can edit the drug prescribed, reduction rate, or duration of projected intervals in AT Planner (see Figure 3a), which updates the projection further. (b) To aid in medication-taking and prescription, AT Planner automatically generates notes for patient and pharmacy for the selected intervals.

AT Planner allows providers to flexibly adjust taper schedules to fit their clinical practice. Providers can modify the automatically-generated schedule to fit their regimens. When any row is clicked in the table, a modal window pops up and lets providers change the type of medication, next dosage, projection mode (linear or exponential), length of intervals, and goal dosage (see Figure 4 (a)). When the changes are submitted, AT Planner repopulates the selected row and all following rows with updated dosages based on the changed condition. For example, in the previous example, if a provider changed the third interval’s dosage from 37.5 mg to 25 mg and instead increased the duration of intervals from 2 weeks to 4 weeks, those changes are projected to the rest of the schedule to lengthen all subsequent intervals and adjust the subsequent reduction rate. AT Planner then saves the projected schedule to a patient profile in the tool (we note that we did not save the projected schedules in practice, and schedules would disappear upon reloading AT Planner). We envision that when a patient returns for a follow-up consultation, the provider could revisit the projected schedule to make iterative adjustments in light of patients’ reactions to the taper.

#### 6.2.4 Generating Notes for Communication.

To allow providers to communicate the prescriptions with pharmacies and patients, AT Planner automatically creates notes based on the projected schedule and selected intervals in plain text (Figure 4 (b)). The notes for patients provide instructions on how to take the prescribed medication in each interval, such as the combination of each strength of pills. The notes for pharmacy give a brief version of patient instructions with a subtotal and a total number of each strength of pills to be prescribed. We envisioned ways that AT Planner could help providers connect the plans to existing healthcare systems and communicate them to their patients if they were adopted in practice. Using the copy-to-clipboard feature, providers could copy and paste the notes to their EMR systems or share them with patients. Providers could also send the information to patients via email, a patient-facing app, or print a PDF file (Figure 4 (b)). Note that we did not implement these sharing methods; instead, we used the buttons to invite conversations about their utility in our interviews.

## 7 FEEDBACK STUDY FINDINGS

We found that providers’ tapering planning practices were influenced by interpersonal and infrastructural constraints, resulting in taper plans which balanced conflicting needs. Providers’ feedback on AT Planner also pointed to desires for different types of support through technology based on their clinical experience in tapering antidepressants.

### 7.1 Impact of Interpersonal and Infrastructural Needs

Providers’ taper planning processes often involved careful consideration of the needs of individual patients, pharmacies, and insurance companies. Therefore, the tapering plans that providers developed were often the result of balancing conflicting interpersonal and infrastructural needs.

#### 7.1.1 Consideration of Patients’ History, Financial Circumstances, and Health Literacy.

Consistent with the findings from the formative study, providers indicated that they consider various factors about the patients they prescribe for when developing taper plans. As mentioned in Section 5.1, providers took patients’ overall health and medication history into account, such as administering more careful tapering regimens if a patient had shown sensitivity towards withdrawal symptoms previously or had been on an antidepressant for years. For example, PS3 mentioned that he would prescribe alternating doses rather than tapering down to a fixed dose right away for patients who have been on antidepressants for an extended period of time (e.g., ten years). If providers expected that patients might experience withdrawal symptoms, they would often prescribe extra pills in case they cannot tolerate the dose changes. PS1 noted: *“If I’m anticipating a rocky taper, they might have to back up and go to a higher dose again.”* PS1 thus added eight pills to the total prescription that AT Planner calculated based on the doses prescribed and the duration of each interval.

Patients’ financial circumstances were also an important consideration for providers. GP4 worked for a federally qualified health center focused on treating underserved populations, which impacted his taper regimens. Many of his patients did not have insurance or were on Medicaid, which is operated by the U.S. federal and state governments to provide health coverage for people living in poverty [53]. When tapering such patients, GP4 would opt for filling the fewest prescriptions versus using the best tapering strategies to accommodate their financial circumstances: *“They are really averse to having to spend extra money [getting another prescription]. […] What I tend to do for uninsured patients is to try to work with what they’ve got. I would get them to break it [tablet] in half for a couple of weeks and then get them to take one every other day or every third or fifth day. It’s not so regimented, but I would rather do what will be helpful for them than have them not be interested in following directions.”*

In addition, providers also considered patients’ health literacy when developing tapering plans, particularly when using complex tapering strategies such as cutting a tablet in quarters (GP3, PS4), measuring liquid formulations with a syringe (PS2), alternating between different dosages (PS3), or taking different strengths of pills at a time (PS1, GP1, PS3). For instance, PS4 mentioned that she would instruct patients to cut pills only when she was confident they would be able to: *“You have to make sure the person is going to be able to cut the pills and be reliable about it. A lot of people just forget, won’t know the pill cutter, or won’t cut them in half.”* GP3 similarly could give complex instructions such as cutting pills into quarters for some patients, but not others: *“We always want to be aware of their abilities for safety for this [cutting tablets in quarters]. […] It’s very hard to cut a pill into quarters, but if they are very high-functioning, and have a very good health literacy, then I would think they would go do this.”* Although GP3 understood that prescribing pills into quarters is not standard practice, he wanted AT Planner to support it. PS2 was also mindful of patients’ health literacy when deciding what drug formulation and how much dose to prescribe. Since measuring liquid with a syringe could be a challenging task for patients, PS2 did not like the precision involved in liquid prescriptions suggested by AT Planner even though it mathematically fit his taper plan: *“I’m not going to tell them to measure all this, like 7.5 ml, 5.6 ml, over the next six months”* To minimize asking patients to precisely measure liquid, PS2 said he would start the taper with pills and change to liquid formulations once the patient gets down to lower doses, such as 25 mg.

#### 7.1.2 Constraints by Pharmacies and Insurance Companies Influencing Clinical Decision Making.

Providers also often considered the constraints that pharmacies and insurance companies would put on what they were able to prescribe, including requiring authorization processes (PS2, PS5), altering prescriptions (PS3, GP1), or rejecting filling medications (PS1, PS2, PS4, PS5). They frequently mentioned they would face the constraints when sending non-standard prescriptions such as multiple strengths of pills at a time and felt that AT Planner’s taper schedules which involved such prescriptions would not be feasible in their practice. PS3 said: *“The pharmacy and many health plans will reject that. They won’t allow the patient to get 40 [mg] and 20 [mg].”* PS5 similarly thought, *“If I put multiple prescriptions for 100 mg, 50mg, and 25mg, they’re like, ‘what?’ Then I usually get a kickback”* PS4 added that some health plans also restrict prescribing high quantities of smaller sizes of pills: *“I tend to like prescribing the fewest amount of different pills. But sometimes, insurance companies will say there’s a quantity limit. Some insurances may not let me prescribe just four 5 mg pills. They’ll say you have to prescribe a higher dose, like one 20 mg pill. A lot of times, the cost per pill is pretty similar, so it is cheaper to get the highest dose possible.”* PS4 thus valued that AT Planner suggested the minimum total count of pills in the projected schedules. Furthermore, providers said some health plans would not cover particular registered doses, and prescriptions generated by AT Planner might not be useful in those cases. PS5 explained: *“Some doses might not be covered [by patients’ health plans]. I’ve run into problems prescribing Prozac 30 mg. 20 [mg] is covered. 10 [mg] is covered. But 30 [mg] is not. Then I’ll have to jigsaw puzzle the next dose.”*

Providers described that pharmacies and insurance companies also had particular volumes of prescriptions that they preferred prescribing. PS2 explained the reasons: *“What they [some pharmacies] are saying is if you’re just prescribing the same drug and if it’s not changing over month by month, it’s most convenient for the patients, and they [insurance companies] save a little money to do a 90-day prescription.”* PS3 similarly described: *“For most of the insurances, I would probably send 90 pills. What often happens is that I’ll write a prescription for 30-day, for example, and I’ll get a message back the next day asking if the patient can get a three-month supply because they prefer that.”* Therefore, he perceived AT Planner using the taper plan to automatically calculate the number of pills to be prescribed would be ineffective: *“Here, it came up with 72 pills because of the next appointment. But if it goes to the technician who receives that prescription, they’re going to think that’s odd. I may get a message in the evening asking to confirm if I only wanted 72 pills. So I’m going to tell the patient I want you to take 20 mg until our next appointment, but I would never prescribe 72 pills.”*

Since different pharmacies and insurance companies had varied constraints and expectations, providers would often try to prescribe and see what happens with individual cases. PS5 noted: *“I don’t really have a good way of knowing it beforehand. I usually just submit it, and then something happens, like either the patient can’t pick it [medications] up, or the pharmacy will try calling us”* To mitigate the providers’ burden, PS4 mentioned that her EMR system recently introduced a new feature that provides information about the quantity limit of patients’ insurances: *“They just brought out a new tool where we can do a payment estimate using their insurance. Just how much it is or if there’s any quantity limit, like 90 tabs per month”* However, it did not provide information about other constraints that the health plans might have. Therefore, she said she would still have to *“wait until you get a rejection by something”*

#### 7.1.3 Accommodating and Circumventing Constraints.

Even though providers were generally aware of the benefits of administering a gradual taper and making prescriptions easier for patients to follow, pharmacies and insurance companies imposed various constraints that impede such strategies. Taper planning often involved non-standard prescriptions such as making frequent dose changes, requiring providers to communicate with pharmacists to clarify. GP1 explained: *“When you have the same medication with two different doses at the same time, they don’t know if it was a mistake or was on purpose. They’ll call you back and ask, ‘Did you want to do 40 [mg] or 20 [mg]?”‘* PS5 also noted the same issue: *“I’ve had to make a lot of calls to pharmacies, to clarify what the goal with all these dosages are.”* Providers perceived that such practices were time-consuming and interfered with their workflow in developing tapering plans. GP1 said: *“Sometimes it’s really frustrating. […] I have to call them, and it takes another 10 minutes”* Providers would sometimes take extra steps to prevent time-consuming communication with pharmacies. GP1 noted: *“So what I found helpful is to include in the note to the pharmacy that the patient needs to take ‘20 [mg] plus 10 [mg].’ And then they know that I’m meaning to send those two at the same time. It’s an extra step, but it’ll definitely save you a phone call and a delay in getting the medication to the patient”* GP4 would also call the pharmacy when sending prescriptions for tapering, expecting he would get phone calls from pharmacists otherwise: *“If it’s going to be something more complicated, I will usually just call the pharmacy after I send the prescription over and talk with the pharmacist so that they understand what my rationale is. That way, they’re not going to be calling me constantly.”* He expected that sharing notes for pharmacies generated by AT Planner would allow him to save such phone calls: *“With this [notes generated by AT Planner], they’d understand what you’re doing. So it saves the hassle of a phone call”*

Notably, providers often developed workarounds for the constraints of pharmacies and insurance companies. Providers sometimes sent pharmacies prescriptions that did not match with what they actually wanted patients to do. For example, PS3 illustrated how he had worked around the pharmacies’ practices when he prescribed alternating doses as tapering strategies, explaining: *“What I prescribe sometimes doesn’t translate to what’s being filled. They [pharmacies] may make adjustments with the pill size based on my prescription. So I would say take 20 [mg] twice a day, one in the morning and the other at night, in the prescription [being sent to the pharmacy]. And I’ll tell the patient that I still want them to take both [two 20mg pills] all at once every other day. I’m doing this for the purpose of the pharmacy giving them 20 [mg] for sure. That way, when I send that prescription, they [pharmacies] can’t automatically give her 40[mg]. This is a way to ensure she could do 40 [mg], 20 [mg], 40 [mg], 20 [mg] without any interference”* Therefore, he disliked that AT Planner only enabled daily prescriptions, even though a daily prescription is standard practice for antidepressants [19]. PS1 would similarly work around constraints by prescribing a big supply of the lowest available dose if he knew that the patient’s health plan and pharmacy would push for a 90-day supply: *“What I would probably do is to give them a big honking supply of 10 mg [pills]. And then just do [taper] it in increments of the 10 mg tablets. So we could give ‘a 90-day supply’ to do a month-long taper. I’ll just write out ‘Take three [10 mg] tablets daily’ in the patient instructions without that being the record. That’s one way around it”* PS1 therefore wished that AT Planner could suggest different combinations of pills to get a certain dose, so that he could select from them in light of other constraints: *“If this [AT Planner] had multiple options, I could say ‘I know this insurance company. I know this patient, then actually pick and choose from those options”*

Furthermore, providers often adjusted the complexity, speed, and combinations of pills of taper schedules to accommodate infrastructural constraints and patient factors. For example, PS2 would typically prescribe and reuse a single strength of pill to avoid a time-consuming authorization process with insurance companies and pharmacies. PS2 explained: *“If you started at 200 [mg] and you want to reduce to 50 [mg], I’m probably not gonna want to deal with these 25 mg tablets. I might just say take one and a half of 100 [mg] for the next month, 100 [mg] the following month, and then half of 100 [mg]. So that way, you don’t have to deal with the pharmacy. I’m not necessarily prescribing separate 25 mg tablets, and having the insurance company give me a hassle about doing authorization. Ergh…forget about it”* As a result, PS2 wanted to be able to administer a faster taper than what AT Planner suggested, but would more gradually taper if the circumstances required it: *“If the patient has demonstrated that sensitivity to withdrawal symptoms and they want to use the smaller doses to have smaller steps, I would definitely do a longer taper.”*

### 7.2 Desiring Flexibility vs. Automation Based on Clinical Experience

Providers’ training and level of experience influenced their desires for tool support. Psychiatrists’ ample experience in tapering antidepressants led them to desire greater flexibility in the tool to support their current strategies. Conversely, general practitioners’ relative lack of experience in tapering antidepressants led them to wish for a greater degree of automation to guide their taper planning.

#### 7.2.1 Appreciation of Flexibility over Automation.

In general, psychiatrists perceived that AT Planner was a flexible tool which could support their complex tapering regimens. For example, psychiatric providers valued that they were able to flexibly change the reduction mode between linear and exponential taper. While PS2 thought *“most people will be fine with just linear taper”* he saw value in administering exponential taper for patients who might be vulnerable to withdrawal symptoms: *“Doing this [exponential taper] would allow you to help the small percentage of people avoid very unpleasant withdrawal symptoms”* Psychiatrists also appreciated that AT Planner allowed them to flexibly adapt taper schedules, especially in later phases of the taper, where patients are more likely to experience withdrawal symptoms. PS1 said, *“Oftentimes, the end of the taper is the hardest. That’s when we want to be able to break it down into smaller and smaller increments.”* PS4 agreed with the need for adjusting taper schedules towards the end: *“There are so many individualized changes that occur that I don’t know [in advance]. It’s hard to plan for the entire taper because so much of it depends on how people react to parts of the taper.”* Therefore, he perceived that AT Planner supporting adjustments would allow them to react to patients’ experiences.

Some psychiatrists desired even greater flexibility to support their complex practices relative to what AT Planner provided. For example, PS3 often instructed his patients to alternate doses every other day: *“In the next two months, the patient is going to be on 40 mg one day and 20 mg the other day.”* He would also prescribe a higher dose on a certain week for patients with other health conditions: *“There could be a premenstrual dysmorphic disorder where their depression is more pronounced one week before they have menstruation. I would say we want to keep that one week at 40 [mg], but the other weeks I’m comfortable with 20 [mg].”* PS4 wished that AT Planner supported prescribing tablets and capsules together: *“We would want to have more options, like being able to combine capsules and tablets. […] Because there’s not a lot of flexibility with capsules, sometimes for the end of the taper, I actually mix it up and add some of the instant release tablets in it.”* In addition, PS1 hoped that he could flexibly change the combinations of pills to gain a certain dose: *“If this [AT Planner] had multiple options to select from one 30 [mg] tablet, or one 10 [mg] and one 20 [mg] tablet, or three 10 mg tablets, [I’d] then actually pick and choose from those options.”*

Psychiatrists generally did not feel that the automatic generation of the projected schedule would be useful, since they were confident in their own ability to support tapering antidepressants. PS3 noted, *“As a long-term psychiatrist, this is easy stuff. It really is. Depression is our primary area of treatment. We know all the antidepressants. When to introduce them, how to decrease them.”* PS2 similarly felt schedule generation would mostly not help him: *“The math isn’t as complicated when you are just doing each step a month.”* However, he thought automatic schedule generation could be useful when schedules become complex: *“The most complicated ones are where you make a change each week or every other week because then it starts to get very tricky with the number of pills you’re getting in a monthly prescription. Then, I have to sit here and do some mental math to make sure the total number [of pills] is right. That’s when it’s most helpful to have something like this. (PS2)”* Similarly, PS4 similarly perceived that it would be helpful for more complicated tapering strategies such as cross-tapering: *“I don’t know if I would need this [AT Planner] for just tapering off one medication because I can do it easily in our EMR. It’s harder if I’m doing cross-tapering. In that case, it would be more helpful.”*

#### 7.2.2 Desiring Automation Relative to Flexibility.

Compared to psychiatrists who generally desired flexibility in technology support for taper planning, general practitioners desired a greater degree of automation. GP3 appreciated that AT Planner automatically generated aspects of the taper schedule that his EMR system did not provide: *“With the current EMR system, there’s no automation. There’s no way of just saying taper by 50% every two weeks until you’re done. So having this functionality for a taper would be very useful.”* GP1 also perceived that AT Planner would make his practice of planning tapers more efficient: *“I think it helps because you don’t have to type it out. […] Otherwise, you have to write an individual prescription for each step by clicking a bunch of buttons in the EMR system.”* Similarly, GP4 valued that using AT Planner would help him save time creating multiple prescriptions for planning for tapers: *“Honestly, it’s a real hassle to have to do that because prescriptions are discrete data points in our EMR. So I just type everything out [when tapering], and that’s time-consuming. This [AT Planner] probably even took 10 seconds. You have all this stuff in each prescription. I can just copy and paste. It’s just so much easier.”* Although most psychiatrists felt confident in their ability to schedule tapers on their own, a few agreed with the general practitioners and appreciated AT Planner’s automation. PS1 perceived that the projection of tentative taper schedules could improve efficiency in his practice: *“I love that the calculations were behind the scene. It was really neat to see the prediction that I wanted over here. […] It saves me the time of having to calculate it and thinking of the dates. It’s just handy enough.”* Likewise, PS5 thought AT Planner’s automatic generation of instructions would be easier for her to write prescriptions than her current EMR system which does not align well with tapering medications: *“The way our EMR system works, if I wanted to give them like a 100 mg pill, then a 50 [mg] pill, and then a 25 [mg] pill, those would be all kind of separate prescriptions that I would have to put in. It’s kind of a pain. […] It [AT Planner] generates all those instructions that make my life easier, rather than typing it all out.”*

Furthermore, general practitioners wished AT Planner incorporated an even greater degree of automation to guide their taper plans. For example, GP1 wished AT Planner indicated what a standard taper for a drug would be: “*I want it to be more automatic somehow. Instead of specifying a current and upcoming dose, you might want to have something automatically pops up saying this is the standard taper*.” Similarly, GP3 also hoped that a system could provide evidence-based recommendations and prognosis of tapering schedules: “*If there’s some evidence base behind it to inform clinical decision making, that could be helpful. What does the evidence say about how quickly you should take somebody off of Paxil 20 mg, and then it would tell you to do two weeks or four weeks. […] Or it can be used for people to input what they are doing and then the system can spit out the likelihood of withdrawal symptoms, etc*.” General practitioners were particularly less confident about dealing with complex tapering strategies, such as cross-tapers. GP1 noted: “*For cross-taper, as a primary doctor, we are not as comfortable doing this because we don’t do this all the time. It would be helpful to get more guidance on what a normal taper is*.” GP3 also resonated with the need for technological guidance on cross-tapering: “*I think the more complex ones where a tool like this can be more helpful are when a patient wants to go from Paxil to Prozac. What is the best way? Is there some sort of cross-taper where you just discontinue one and start the other?*”

The finding on primary care providers’ needs for automatic guidance through a tapering support tool is consistent with the needs that other formative study participants mentioned. In the formative study, all general practitioners and nurse practitioners mentioned their desire to gain guidance through tapering support tools. For instance, NP2 stated: “*If you indicate tapering in certain conditions and there’s maybe standard tapering doses, that would be helpful*.” NP1 similarly expressed the desire to gain automated recommendations: “*Ideally, I would like a recommendation of tapering based on current dosage, length of time patient has been on medication, age, and average health of the patient. If I plug those in and be given recommendations of how to decrease it, such as decreasing by 50% every two weeks, that would be helpful*.”

## 8 DISCUSSION

Our findings from designing AT Planner and getting feedback from providers on its design suggest opportunities for improving clinical tools to support planning practices. We found providers played a role in balancing different interpersonal and infrastructural constraints, which might have been exacerbated by a lack of established clinical guidelines and the requirements of longitudinal planning. Based on the findings, we discuss how technology can better support providers in balancing the influencing constraints on developing care plans. We also found that providers with varying levels of experience in tapering antidepressants had different design needs for clinical decision support tools. Providers with more experience were likely to desire a flexible tool to support their current practice, whereas providers with less experience were likely to seek automated guidance from technology. We suggest that providers’ varying levels of experience need to be carefully considered in the design of clinical decision support tools. Lastly, we consider the opportunities and challenges of clinical decision support tools operating outside the EMRs in research and in practice.

### 8.1 Developing Flexible Planning Tools to Assist Providers in Balancing Influencing Constraints

Through this study, we found that providers’ planning practices for tapering antidepressants were under various influencing constraints by individual patients, pharmacies, and insurance companies. In our work, providers played a significant role in balancing such constraints. Providers are often engaged in complex evaluations on what combinations of pills they should prescribe to meet individual pharmacies’ and health plans’ constraints while ensuring the instructions are not too complex for patients to follow nor not affordable for patients. Prior work has emphasized the importance of considering sociotechnical aspects in the design of clinical support tools [9, 10, 34, 57, 64]. Consistent with prior work, our findings suggest that clinical decision support tools should be mindful of other interpersonal and infrastructural factors in play [46]. We posit the challenges in balancing sociotechnical factors are particularly likely to emerge in longitudinal care planning than in one-time clinical decision-making contexts. In our work, longitudinal taper planning required multiple dose changes over time and iterative adjustment of schedules. Adapting plans to provide care that was responsive and accommodating to patient needs often required providers to make non-standard prescriptions, such as combining different strengths of pills or many small pills that pharmacies or insurance companies may not allow.

Prior medical literature has frequently mentioned that providers often used workarounds to circumvent the requirements of EMR systems [9, 45, 57]. These workarounds mainly occur when the sociotechnical contexts where the systems are used have not been accounted for in the design [9, 45]. Our participants similarly indicated using workarounds to circumvent the interpersonal and infrastructural constraints. Specifically, we suspect that the lack of standardization in clinical guidelines around tapering antidepressants is likely to have led providers to seek out loopholes in EMR systems because the pharmacies and insurance companies they worked with lacked policies and protocols that align with the care regimens required for tapering as a result. Prior work on EMR systems has suggested allowing space for providers to indicate their needs to other groups [61]. Extending this, our study suggests the value of flexibility in clinical decision support tools to accommodate providers’ needs, particularly around longitudinal planning and a lack of standardized guidelines. However, addressing the larger problem of getting care regimens recognized by other stakeholders requires creating and formalizing guidelines through clinical trials. Before clinical guidelines are established, technology can help support flexibility in developing care plans for patients.

### 8.2 Different Levels of Experience Impacting the Design Needs for Clinical Decision Support Tools

We found that providers with relatively less experience, such as primary care providers, often faced challenges in developing taper plans due to their unfamiliarity and lack of clinical guidance. Therefore, they desired different types of support from what providers with more experience, such as psychiatrists, desired. While the most experienced psychiatrists were often skeptical that technology-driven decision support would even be helpful and preferred to rely solely on their own experience, primary care providers desired more automated guidance to offload the decision-making burden to technology. Many health conditions, including diabetes, HIV, and depression, are treated in both primary and specialist care settings. While primary care has benefits in providing continuous and accessible care for patients throughout their life course [37, 72], prior work frequently mentioned primary care providers’ concerns about knowledge and experience with regimens that can be complex or risky [37, 41, 72]. Although both groups might face challenges in developing care regimens with a lack of clinical guidance, specialists are more likely to develop their own regimens over time based on their empirical knowledge resulting from many trials and errors; on the other hand, primary care providers are less likely to feel confident about developing their own regimens due to their limited experience in specific domains relative to specialists [41, 58].

This finding indicates that providers’ varying levels of experience should be carefully considered in the design of clinical decision support tools. Prior work on clinical decision support tools suggested the potential of AI-powered systems in providing data-driven recommendations [8, 13, 29, 49–51]. This approach could help providers with less experience navigate the challenges of developing care regimens with a lack of clinical guidance. On the other hand, because providers with more experience are likely to have developed their unique regimens with the lack of standard clinical guidelines, providing automatic recommendations without the ability to make adjustments might make them feel that their expertise is not recognized [43, 73, 78]. Providers with more experience might instead benefit the most from a flexible tool to support their regimens involving complex strategies and iterative adjustments. Or alternatively, workflows could enable experienced providers not to engage with tool support at all. A challenge in this space is that it is difficult to design a single tool to provide both automated recommendations and flexibility to support various regimens of providers. When possible, understanding the experience levels and needs of the target providers before designing new clinical decision support tools can help tailor them to the audience. In cases where it is not possible to tailor such tools to a specific target audience, we should consider developing tools that offer guidance only when needed or requested but let providers leverage their own expertise.

### 8.3 Opportunities and Challenges of Clinical Decision Support Tools Operating in and outside the EMRs

Though we did not aim to evaluate the clinical efficacy of AT Planner, its development led us to reflect on the relative advantages of prototyping clinical decision support technology in and outside of EMRs. Our motivations for designing AT Planner as an online tool outside of an EMR were predominantly practical. Doing so allowed us to implement the design without having to be tied to a specific EMR system for participant recruitment (e.g., recruiting only participants who work in medical systems using Epic), and avoiding regulatory needs around developing an add-on to an EMR tool and software installation on computers in hospitals and clinics. This decision greatly reduced prototyping time and participant recruitment, enabling us to efficiently evaluate our design ideas and contribute a broader understanding around clinical decision support tools. We thus see significant advantages for HCI researchers to prototyping clinical decision support tools outside the EMRs for developing an understanding of design approaches.

Despite the benefits of implementing tools operating outside the EMRs, significant challenges emerge when considering the utility of the approach in field deployments or extending from a design idea to clinical adoption. A main challenge is that the barrier to provider adoption remains high. Previous studies primarily highlighted the need for clinical decision support tools to be integrated into the existing health care systems, such as EMRs, to minimize interruptions and fit into providers’ workflow [34, 38, 77, 78]. Similarly in our study, even though AT Planner generated output for prescriptions that could theoretically be copied into their EMR, providers often expected using the tool would involve additional work for them to double-chart prescriptions or other patient information. They hoped that tools like AT Planner would be integrated into EMRs, and expected that such tools are unlikely to be used in clinical environments otherwise.

Another significant challenge mentioned by several HCI studies [15, 79, 80] is avoiding the use of any personal health data in the tool itself under the medical data regulations such as the Health Insurance Portability and Accountability Act (HIPAA) in the U.S. and the General Data Protection Regulation (GDPR) in the European Union. One model providers in our study frequently leveraged was using online resources to look up information about antidepressants (e.g., GoodRx [26]). Designing clinical decision support tools to similarly serve purely as resources, where inferences or plans can be developed and exported to EMRs, is one potential opportunity for standalone technology. However, this is often not possible for many kinds of tools, such as AI tools which aim to aid with the interpretation of patient health data.

In a domain like tapering antidepressants where medical advice is still evolving, operating outside the EMR enables tools to more quickly respond to the evolving medical literature, such as by integrating recommendations as clinical trials are published. Although tools operating outside EMRs can more easily integrate advice, they lack regulation important to ensuring the advice is clinically supported. Participants in our formative interviews, particularly primary care providers, frequently mentioned desiring advice on effective taper plans for particular medications. However, we intentionally opted not to incorporate such advice for a few reasons. We sought to avoid going beyond our primary area of expertise, as the medical community is in the phase of conducting individual trials, and tapering recommendations have not yet been formalized into national guidelines. We were also concerned that the advice offered by AT Planner could be subject to regulation in future stages of research, reducing our ability to quickly evaluate the design ideas. However, opting not to include advice came at a cost to the needs of providers, particularly those with less experience with taper planning. Trading off the relative benefits of operating quickly outside of medical record systems with operating carefully inside those systems warrants further consideration in future projects.

## 9 CONCLUSION

Through designing and evaluating clinical decision support for tapering antidepressants, we found providers’ planning practices were often influenced by interpersonal and infrastructural constraints and clinical experience influenced their design needs. Allowing some loopholes in clinical tools can be valuable for navigating infrastructural barriers, particularly in domains with a lack of standardized guidelines. Providers with more experience desire flexibility in decision support systems, while providers with less experience appreciate automated guidance from technology. Therefore, providers’ varying levels of experience should be carefully considered in the design of clinical decision support tools. Lastly, we suggest that developing decision support tools which operate outside EMRs can allow HCI researchers to quickly implement the design and more quickly respond to evolving medical literature. However, the barrier to provider use of external tools remains high, which presents challenges when conducting field deployments or extending to clinical adoption.

## Supplementary Material

Video demonstrating AT Planner’s functionality and how it can be used.

Study protocols and term glossary.

## Figures and Tables

**Table 1:** Participant demographics and study participation. PS# denotes psychiatrist or psychiatric resident. GP# denotes general practitioners in Family Medicine, and NP# denotes nurse practitioners.

Participant ID	Years post-residency	Study participation	
		Formative	Feedback
PS1 (M, 33)	2	✓	✓
PS2 (M, 50)	18	✓	✓
PS3 (M, 59)	30	✓	✓
PS4 (F, 37)	5		✓
PS5 (F, 29)	4th-year resident		✓
NP1 (F, 36)	1	✓	
NP2 (M, 37)	2	✓	
NP3 (M, 41)	9	✓	
GP1 (M, 33)	3	✓	✓
GP2 (F, 35)	11	✓	
GP3 (M, 37)	7		✓
GP4 (M, 59)	26		✓

## CCS CONCEPTS

**• Human-centered computing → Human computer interaction (HCI)**; User studies; **• Applied computing → Health informatics**; **• Information systems** → Decision support systems.

## Footnotes

1 A full description of relevant medical terms can be found in the glossary in our supplemental materials.

## References

[1] Aarts Jos, Ash Joan, and Berg Marc. 2007. Extending the understanding of computerized physician order entry: Implications for professional collaboration, workflow and quality of care. International Journal of Medical Informatics 76, Suppl. 1 (2007), S4–s13. 10.1016/j.ijmedinf.2006.05.009 16798068

[2] Abu-Geras Dana, Hadziomerovic Dunja, Leau Andrew, Nazim Khan Ramzan, Gudka Sajni, Locher Cornelia, Razaghikashani Maryam, and Lim Lee Yong. 2017. Accuracy of tablet splitting and liquid measurements: an examination of who, what and how. Journal of Pharmacy and Pharmacology 69, 5 (2017), 603–612. 10.1111/jphp.12671 28028813

[3] Substance Abuse and Mental Health Services Administration. 2019. Key substance use and mental health indicators in the United States: Results from the 2018 National Survey on Drug Use and Health. HHS Publication No. PEP19-5068, NSDUH Series H-54 170 (2019), 51–58. https://www.samhsa.gov/data/

[4] Agapie Elena, Chinh Bonnie, Pina Laura R., Oviedo Diana, Welsh Molly C., Hsieh Gary, and Munson Sean A.. 2018. Crowdsourcing Exercise plans aligned with expert guidelines and everyday constraints. Conference on Human Factors in Computing Systems - Proceedings 2018-April (2018), 1–13. 10.1145/3173574.3173898

[5] Agapie Elena, Colusso Lucas, Munson Sean A., and Hsieh Gary. 2016. Plan sourcing: Generating behavior change plans with friends and crowds. Proceedings of the ACM Conference on Computer Supported Cooperative Work, CSCW 27 (2016), 119–133. 10.1145/2818048.2819943

[6] Agmon Maayan, Zisberg Anna, Gil Efrat, Rand Debbie, Gur-Yaish Nurit, and Azriel Mary. 2017. Adult Utilization of Psychiatric Drugs and Differences by Sex, Age, and Race. JAMA Internal Medicine 177, 2 (2017), 272–274. 10.1001/jamainternmed.2016.7266 27918776

[7] American Psychiatric Association. 2010. Treatment of Patients With Major Depressive Disorder Second Edition APA. Psychiatric Services April (2010), 1–78.

[8] Beede Emma, Baylor Elizabeth, Hersch Fred, Iurchenko Anna, Wilcox Lauren, Ruamviboonsuk Paisan, and Vardoulakis Laura M.. 2020. A Human-Centered Evaluation of a Deep Learning System Deployed in Clinics for the Detection of Diabetic Retinopathy. Conference on Human Factors in Computing Systems - Proceedings (2020), 1–12. 10.1145/3313831.3376718

[9] Berg Marc. 1999. Patient care information systems and health care work: A sociotechnical approach. International Journal of Medical Informatics 55, 2 (1999), 87–101. 10.1016/s1386-5056(99)00011-8 10530825

[10] Berg Marc, Aarts J, and Van der Lei J. 2003. ICT in Health Care: Sociotechnical Approaches. Methods of Information in Medicine 42, 4 (2003), 297–301. 10.1055/s-0038-1634221 14534625

[11] Braun Virginia and Clarke Victoria. 2006. Using thematic analysis in psychology. Qualitative Research in Psychology 3, 2 (2006), 77–101.

[12] Bull Scott A., Hunkeler Enid M., Lee Janelle Y., Rowland Clayton R., Williamson Todd E., Schwab Joseph R., Hurt Stephen W., Gonzalez Lydia, and Demers Denyse. 2002. Discontinuing or switching selective serotonin-reuptake inhibitors. Annals of Pharmacotherapy 36, 4 (2002), 578–584. 10.1345/aph.1A254 11918502

[13] Cai Carrie J., Reif Emily, Hegde Narayan, Hipp Jason, Kim Been, Smilkov Daniel, Wattenberg Martin, Viegas Fernanda, Corrado Greg S., Stumpe Martin C., and Terry Michael. 2019. Human-centered tools for coping with imperfect algorithms during medical decision-making. Conference on Human Factors in Computing Systems - Proceedings (2019), 1–14. 10.1145/3290605.3300234 arXiv:1902.02960

[14] Cai Carrie J., Winter Samantha, Steiner David, Wilcox Lauren, and Terry Michael. 2019. “Hello Ai”: Uncovering the onboarding needs of medical practitioners for human–AI collaborative decision-making. Proceedings of the ACM on Human-Computer Interaction 3, Cscw (2019). 10.1145/3359206

[15] Chia Fang Chung Kristin Dew, Cole Allison, Zia Jasmine, Fogarty James, Kientz Julie A., and Munson Sean A.. 2016. Boundary negotiating artifacts in personal informatics: Patient-provider collaboration with patient-generated data. Proceedings of the ACM Conference on Computer Supported Cooperative Work, CSCW 27 (2016), 770–786. 10.1145/2818048.2819926 PMC5432205 28516171

[16] Chung Chia-Fang, Wang Qiaosi, Schroeder Jessica, Cole Allison, Zia Jasmine, Fogarty James, and Munson Sean. 2019. Identifying and Planning for Individualized Change: Patient-Provider Collaboration Using Lightweight Food Diaries in Healthy Eating and Irritable Bowel Syndrome. Proceedings of the ACM on Interactive, Mobile, Wearable and Ubiquitous Technologies 3, 1 (2019), 1–27. 10.1145/3314394 31080941 PMC6504841

[17] Daskalova Nediyana, Metaxa-Kakavouli Danaë, Tran Adrienne, Nugent Nicole, Boergers Julie, McGeary John, and Huang Jeff. 2016. SleepCoacher: A personalized automated self-experimentation system for sleep recommendations. UIST 2016 - Proceedings of the 29th Annual Symposium on User Interface Software and Technology (2016), 347–358. 10.1145/2984511.2984534

[18] Davies James and Read John. 2019. A systematic review into the incidence, severity and duration of antidepressant withdrawal effects: Are guidelines evidence-based? Addictive Behaviors 97, September 2018 (2019), 111–121. 10.1016/j.addbeh.2018.08.027 30292574

[19] DeVane C.Lindsay. 1994. Pharmacokinetics of the newer antidepressants: Clinical relevance. The American Journal of Medicine 97, 6 (dec 1994), S13–S23. 10.1016/0002-9343(94)90359-X 7992822

[20] Elwyn Glyn, Scholl Isabelle, Tietbohl Caroline, Mann Mala, Adrian Gk Edwards Catharine Clay, Légaré France, Van Der Weijden Trudy, Lewis Carmen L., Wexler Richard M., and Frosch Dominick L.. 2013. “Many miles to go.”: A systematic review of the implementation of patient decision support interventions into routine clinical practice. BMC Medical Informatics and Decision Making 13, Suppl. 2 (2013), S14. 10.1186/1472-6947-13-s2-s14 24625083 PMC4044318

[21] Figueiredo Mayara Costa, Caldeira Clara, Reynolds Tera L., Victory Sean, Zheng Kai, and Chen Yunan. 2017. Self-tracking for fertility care: Collaborative support for a highly-personalized problem. Proceedings of the ACM on Human-Computer Interaction 1, Cscw (2017). 10.1145/3134671

[22] Figueiredo Mayara Costa and Chen Yunan. 2020. Patient-generated health data: Dimensions, challenges, and open questions. Foundations and Trends in Human-Computer Interaction 13, 3 (2020), 165–297. 10.1561/1100000080

[23] National Institute for Health Care and Excellence. 2018. Depression in adults: treatment and management Full guideline. May (2018). 35977056

[24] Framer Adele. 2021. What I have learnt from helping thousands of people taper off antidepressants and other psychotropic medications. Therapeutic Advances in Psychopharmacology 11 (2021), 204512532199127. 10.1177/2045125321991274 PMC7970174 33796265

[25] Garg Amit X., Adhikari Neill K.J., McDonald Heather, Patricia Rosas-Arellano M, Devereaux PJ, Beyene Joseph, Sam Justina, and Brian Haynes R. 2005. Effects of computerized clinical decision support systems on practitioner performance and patient outcomes: A systematic review. Journal of the American Medical Association 293, 10 (2005), 1223–1238. 10.1001/jama.293.10.1223 15755945

[26] GoodRx. 2021. Accessed Sep 2nd, 2021. https://www.goodrx.com/

[27] Gorman Paul, Lavelle MB, and Ash JoanS. 2003. Order creation and communication in healthcare. Methods Inf Med 42, February 2003 (2003). 10.1267/meth03040376 14534637

[28] Groot Peter C. and van Os Jim. 2020. How user knowledge of psychotropic drug withdrawal resulted in the development of person-specific tapering medication. Therapeutic Advances in Psychopharmacology 10 (2020), 204512532093245. 10.1177/2045125320932452 PMC7357127 32699604

[29] Gu Hongyan, Huang Jingbin, Hung Lauren, and Chen Xiang Anthony. 2021. Lessons Learned from Designing an AI-Enabled Diagnosis Tool for Pathologists. Proceedings of the ACM on Human-Computer Interaction 5, Cscw1 (2021). 10.1145/3449084 arXiv:2006.12695

[30] Haddad Peter. 1997. the Discontinuation Syndrome rig py Co a du Pr. 58, suppl 7 (1997), 17–21. 9219489

[31] Hallare Jericho and Gerriets Valerie. 2020. Half-life. Accessed Sep 2nd, 2021. https://www.ncbi.nlm.nih.gov/books/NBK554498/

[32] healthcare.gov. 2021. Getting prescription medications. Accessed Sep 2nd, 2021. https://www.healthcare.gov/using-marketplace-coverage/prescription-medications/

[33] Horowitz Mark Abie and Taylor David. 2019. Tapering of SSRI treatment to mitigate withdrawal symptoms. The Lancet Psychiatry 6, 6 (2019), 538–546. 10.1016/s2215-0366(19)30032-x 30850328

[34] Jacobs Maia, He Jeffrey, Pradier Melanie F., Lam Barbara, Ahn Andrew C., McCoy Thomas H., Perlis Roy H., Doshi-Velez Finale, and Gajos Krzysztof Z.. 2021. Designing AI for Trust and Collaboration in Time-Constrained Medical Decisions: A Sociotechnical Lens. Chi (2021), 1–14. 10.1145/3411764.3445385 arXiv:2102.00593

[35] Jacques Emmanuel Reginald and Alexandridis Paschalis. 2019. Tablet scoring: Current practice, fundamentals, and knowledge gaps. Applied Sciences (Switzerland) 9, 15 (2019). 10.3390/app9153066

[36] Jaspers Monique W.M., Smeulers Marian, Vermeulen Hester, and Peute Linda W.. 2011. Effects of clinical decision-support systems on practitioner performance and patient outcomes: A synthesis of high-quality systematic review findings. Journal of the American Medical Informatics Association 18, 3 (2011), 327–334. 10.1136/amiajnl-2011-000094 21422100 PMC3078663

[37] Jeavons D, Hungin APS, and Cornford CS. 2006. Patients with poorly controlled diabetes in primary care: Healthcare clinicians’ beliefs and attitudes. Postgraduate Medical Journal 82, 967 (2006), 347–350. 10.1136/pgmj.2005.039545 16679475 PMC2563795

[38] Kaltenhauser Annika, Rheinstädter Verena, Butz Andreas, and Wallach Dieter P.. 2020. You Have to Piece the Puzzle Together”: Implications for designing decision support in intensive care. DIS 2020 - Proceedings of the 2020 ACM Designing Interactive Systems Conference (2020), 1509–1522. 10.1145/3357236.3395436

[39] Karkar Ravi, Schroeder Jessica, Epstein Daniel A., Pina Laura R., Scofield Jeffrey, Fogarty James, Kientz Julie A., Munson Sean A., Vilardaga Roger, and Zia Jasmine. 2017. TummyTrials: A feasibility study of using self-experimentation to detect individualized food triggers. Conference on Human Factors in Computing Systems - Proceedings 2017-May (2017), 6850–6863. 10.1145/3025453.3025480 PMC5432136 28516175

[40] Kawamoto Kensaku, Houlihan Caitlin A., Andrew Balas E, and Lobach David F.. 2005. Improving clinical practice using clinical decision support systems: A systematic review of trials to identify features critical to success. British Medical Journal 330, 7494 (2005), 765–768. 10.1136/bmj.38398.500764.8f 15767266 PMC555881

[41] Keks Nicholas, Hope Judy, and Keogh Simone. 2016. Switching and stopping antidepressants. Australian Prescriber 39, 3 (2016), 76–83. 10.18773/austprescr.2016.039 27346915 PMC4919171

[42] Kendrick Tony. 2021. Strategies to reduce use of antidepressants. British Journal of Clinical Pharmacology 87, 1 (2021), 23–33. 10.1111/bcp.14475 32656861

[43] Khairat Saif, Marc David, Crosby William, and Sanousi Ali Al. 2018. Reasons for physicians not adopting clinical decision support systems: Critical analysis. JMIR Medical Informatics 20, 4 (2018). 10.2196/medinform.8912 PMC5932331 29669706

[44] Klüber Sara, Maas Franzisca, Schraudt David, Hermann Gina, Happel Oliver, and Grundgeiger Tobias. 2020. Experience matters: Design and evaluation of an anesthesia support tool guided by user experience theory. DIS 2020 - Proceedings of the 2020 ACM Designing Interactive Systems Conference (2020), 1523–1535. 10.1145/3357236.3395552

[45] Koppel Ross, Wetterneck Tosha, Telles Joel Leon, and Karsh Ben-Tzion. 2010. Workarounds to barcode medication administration systems: their occurrences, causes, and threats to patient safety. Journal of the American Medical Informatics Association : JAMIA 15, 4 (2010), 408–23. 10.1197/jamia.M2616 PMC2442264 18436903

[46] Lee Charlotte P., Dourish Paul, and Mark Gloria. 2006. The human infrastructure of cyberinfrastructure. Proceedings of the ACM Conference on Computer Supported Cooperative Work, CSCW (2006), 483–492. 10.1145/1180875.1180950

[47] Lee Jisoo, Walker Erin, Burleson Winslow, Kay Matthew, Buman Matthew, and Hekler Eric B.. 2017. Self-experimentation for behavior change: Design and formative evaluation of two approaches. Conference on Human Factors in Computing Systems - Proceedings 2017-May (2017), 6837–6849. 10.1145/3025453.3026038

[48] Lee Kwangyoung, Cho Hyewon, Toshnazarov Kobiljon, Narziev Nematjon, Rhim So Young, Han Kyungsik, Noh YoungTae, and Hong Hwajung. 2020. Toward Future-Centric Personal Informatics: Expecting Stressful Events and Preparing Personalized Interventions in Stress Management. (2020), 1–13. 10.1145/3313831.3376475

[49] Lee Min Hun, Siewiorek Daniel P., and Smailagic Asim. 2021. A human-ai collaborative approach for clinical decision making on rehabilitation assessment. Conference on Human Factors in Computing Systems - Proceedings Figure 1 (2021). 10.1145/3411764.3445472

[50] Lee Min Hun, Siewiorek Daniel P., Smailagic Asim, Bernardino Alexandre, and Badia Sergi Bermúdez I. 2020. Co-Design and Evaluation of an Intelligent Decision Support System for Stroke Rehabilitation Assessment. Proceedings of the ACM on Human-Computer Interaction 4, Cscw2 (2020). 10.1145/3415227

[51] Levy Ariel, Agrawal Monica, Satyanarayan Arvind, and Sontag David. 2021. Assessing the impact of automated suggestions on decision making: Domain experts mediate model errors but take less initiative. Conference on Human Factors in Computing Systems - Proceedings (2021). 10.1145/3411764.3445522 arXiv:2103.04725

[52] McCabe Joanne, Wilcock Mike, Atkinson Kate, Laugharne Richard, and Shankar Rohit. 2020. General practitioners’ and psychiatrists’ attitudes towards antidepressant withdrawal. BJPsych Open 6, 4 (2020), 1–6. 10.1192/bjo.2020.48 PMC7345735 32552920

[53] Medicaid.gov. 2021. Medicaid. Accessed Sep 2nd, 2021. https://www.medicaid.gov/medicaid/index.html

[54] Middleton B, Sittig DF, and Wright A. 2016. Clinical Decision Support: a 25 Year Retrospective and a 25 Year Vision. Yearbook of medical informatics (2016), S103–s116. 10.15265/IYS-2016-s034 27488402 PMC5171504

[55] Murnane Elizabeth L., Walker Tara G., Tench Beck, Voida Stephen, and Snyder Jaime. 2018. Personal informatics in interpersonal contexts: Towards the design of technology that supports the social ecologies of long-term mental health management. Proceedings of the ACM on Human-Computer Interaction 2, CSCW (2018). 10.1145/3274396

[56] National Institute of Mental Health. 2016. Mental Health Medications. Accessed Sep 2nd, 2021. https://www.nimh.nih.gov/health/topics/mental-health-medications

[57] Niazkhani Zahra, Pirnejad Habibollah, Berg Marc, and Aarts Jos. 2009. The Impact of Computerized Provider Order Entry Systems on Inpatient Clinical Workflow: A Literature Review. Journal of the American Medical Informatics Association 16, 4 (2009), 539–549. 10.1197/jamia.M2419 19390113 PMC2705258

[58] Ogle Nikki R. and Akkerman Shawn R.. 2013. Guidance for the discontinuation or switching of antidepressant therapies in adults. Journal of Pharmacy Practice 26, 4 (2013), 389–396. 10.1177/0897190012467210 23459282

[59] Osheroff Jerome A., Teich Jonathan M., Middleton Blackford, Steen Elaine B., Wright Adam, and Detmer Don E.. 2007. A Roadmap for National Action on Clinical Decision Support. Journal of the American Medical Informatics Association 14, 2 (2007), 141–145. 10.1197/jamia.M2334 17213487 PMC2213467

[60] Papoutsaki Alexandra, So Samuel, Kenderova Georgia, Shapiro Bryan, and Epstein Daniel. 2021. Understanding Delivery of Collectively Built Protocols in an Online Health Community for Discontinuation of Psychiatric Drugs. (2021). preprint available online at https://depstein.net/assets/pubs/apapoutsaki_cscw21.pdf.

[61] Park Sun Young, Pine Kathleen H., and Chen Yunan. 2013. Local-universality: Designing EMR to support localized informal documentation practices. Proceedings of the ACM Conference on Computer Supported Cooperative Work, CSCW (2013), 55–66. 10.1145/2441776.2441786

[62] Patterson Emily S., Cook Richard I., and Render Marta L.. 2002. Improving patient safety by identifying side effects from introducing bar coding in medication administration. Journal of the American Medical Informatics Association 9, 5 (2002), 540–553. 10.1197/jamia.M1061 12223506 PMC346641

[63] Paulose-Ram Ryne, Safran Marc A., Jonas Bruce S., Gu Qiuping, and Orwig Denise. 2007. Trends in psychotropic medication use among U.S. adults. Pharmacoepidemiology and Drug Safety 16, 5 (may 2007), 560–570. 10.1002/pds.1367 17286304

[64] Pontefract Sarah K., Coleman Jamie J., Vallance Hannah K., Hirsch Christine A., Shah Sonal, Marriott John F., and Redwood Sabi. 2018. The impact of computerised physician order entry and clinical decision support on pharmacist-physician communication in the hospital setting: A qualitative study. PLoS ONE 13, 11 (2018), 1–15. 10.1371/journal.pone.0207450 PMC6239308 30444894

[65] Psych RC. 2019. Position statement on antidepressants and depression. Journal of Nursing Studies 49, 10 (2019), 1–29. https://www.rcpsych.ac.uk/docs/default-source/improving-care/better-mh-policy/position-statements/ps04_19---antidepressants-and-depression.pdf?sfvrsn=ddea9473_5

[66] Read John. 2020. How common and severe are six withdrawal effects from, and addiction to, antidepressants? The experiences of a large international sample of patients. Addictive Behaviors 102, July 2019 (2020), 106157. 10.1016/j.addbeh.2019.106157 31841871

[67] Read John, Cartwright Claire, and Gibson Kerry. 2018. How many of 1829 antidepressant users report withdrawal effects or addiction? International Journal of Mental Health Nursing 27, 6 (2018), 1805–1815. 10.1111/inm.12488 29873165

[68] Rohani Darius A., Andrea Quemada Lopategui Nanna Tuxen, Maria Faurholt-Jepsen Lars V. Kessing, and Bardram Jakob E.. 2020. MUBS: A Personalized Recommender System for Behavioral Activation in Mental Health. Conference on Human Factors in Computing Systems - Proceedings (2020), 1–13. 10.1145/3313831.3376879

[69] Schroeder Jessica, Hoffswell Jane, Chung Chia Fang, Fogarty James, Munson Sean, and Zia Jasmine. 2017. Supporting patient-provider collaboration to identify individual triggers using food and symptom journals. Proceedings of the ACM Conference on Computer Supported Cooperative Work, CSCW (2017), 1726–1739. 10.1145/2998181.2998276 PMC5432206 28516172

[70] Scott David M.. 2016. United States health care system: A pharmacy perspective. Canadian Journal of Hospital Pharmacy 69, 4 (2016), 306–315. 10.4212/cjhp.v69i4.1585 27621491 PMC5008427

[71] Shapiro Bryan B.. 2018. Subtherapeutic doses of SSRI antidepressants demonstrate considerable serotonin transporter occupancy: implications for tapering SSRIs. Psychopharmacology 235, 9 (2018), 2779–2781. 10.1007/s00213-018-4995-4 30097698

[72] Sorensen Anna, Le Lisa W., Swami Nadia, Hannon Breffni, Krzyzanowska Monika K., Wentlandt Kirsten, Rodin Gary, and Zimmermann Camilla. 2020. Readiness for delivering early palliative care: A survey of primary care and specialised physicians. Palliative Medicine 34, 1 (2020), 114–125. 10.1177/0269216319876915 31849272

[73] Wang Dakuo, Wang Liuping, and Zhang Zhan. 2021. Brilliant ai doctor in rural clinics: Challenges in ai-powered clinical decision support system deployment. Conference on Human Factors in Computing Systems - Proceedings (2021). 10.1145/3411764.3445432

[74] Warner Christopher H., Bobo William, Warner Carolynn, Reid Sara, and Rachal James. 2006. Antidepressant discontinuation syndrome. American Family Physician 74, 3 (2006), 449–456. 16913164

[75] Wears Robert L. and Berg Marc. 2005. Computer Technology and Clinical Work. Jama 293, 10 (2005), 1261. 10.1001/jama.293.10.1261 15755949

[76] Wolf Nicole J. and Hopko Derek R.. 2008. Psychosocial and pharmacological interventions for depressed adults in primary care: A critical review. Clinical Psychology Review 28, 1 (2008), 131–161. 10.1016/j.cpr.2007.04.004 17555857

[77] Yang Qian, Steinfeld Aaron, and Zimmerman John. 2019. Unremarkable AI: Fiting intelligent decision support into critical, clinical decision-making processes. Conference on Human Factors in Computing Systems - Proceedings (2019), 1–11. 10.1145/3290605.3300468

[78] Yang Qian, Zimmerman John, Steinfeld Aaron, Carey Lisa, and Antaki James F.. 2016. Investigating the heart pump implant decision process: Opportunities for decision support tools to help. Conference on Human Factors in Computing Systems - Proceedings(2016), 4477–4488. 10.1145/2858036.2858373 PMC5101017 27833397

[79] Zhu Haining, Colgan Joanna, Reddy Madhu, and Choe Eun Kyoung. 2016. Sharing Patient-Generated Data in Clinical Practices: An Interview Study. AMIA … Annual Symposium proceedings. AMIA Symposium 2016 (2016), 1303–1312. 28269928 PMC5333267

[80] Zhu Haining, Moffa Zachary J., Gui Xinning, and Carroll John M.. 2020. Prehabilitation: Care Challenges and Technological Opportunities. Conference on Human Factors in Computing Systems - Proceedings (2020), 1–13. 10.1145/3313831.3376594

