# Supplementary material for: Designing Flexible Longitudinal Regimens: Supporting Clinician Planning for Discontinuation of Psychiatric Drugs
Source: Proc SIGCHI Conf Hum Factor Comput Syst. Author manuscript; Available in PMC 2022 Jul 1. (PMC9247721; doi:10.1145/3491102.3502206)
Supplement: Study protocols and term glossary. [file NIHMS1793794-supplement-Study_protocols_and_term_glossary_.zip › Feedback study protocol.pdf]

Feedback study protocol

Introduction

Thank you so much for making time to chat with us. Our research team has been working on this project for designing a clinician-facing support tool for tapering antidepressants. Today, we will ask you to use a web app that we developed to support the planning of tapering antidepressants and get some feedback on whether it aligns with your clinical practice and how we can improve on the design to better support your clinical practice. The interview will last for approximately an hour.

System overview

Let me briefly explain how our system works. AT Planner is focused on supporting iterative planning of tapering SSRIs and SNRIs in an efficient manner. To do that, it creates a projected schedule based on a single interval that you configure. Once you select intervals you would like to prescribe at a time, AT planner generates plain text that can easily be pasted into EHR.

Developing a tapering plan together

First off, could you think of one of your patients or come up with a hypothetical patient trying to or planning to be entirely off of an SSRI or SNRI? If so, could you briefly describe the patient?

We'll ask you to develop a tapering plan for your example patient. The goal of this activity is neither to test your ability to develop tapering plans nor to test the usability of the app. Rather, we are curious to know whether the features are helpful in your clinical practice. So, we'll first ask you to manipulate the app on your own, but we'll be here to help you out too. Please don't hesitate to let us know if you have any questions.

(Share the link through the chat.)

Based on your descriptions, could you make a tapering plan for them using this tool? Again, let us know if you have any questions.

Next, imagine that you find the patient struggling with withdrawal symptoms in their follow-up visit, so you would like to adjust their tapering plan. Please make adjustments to the tapering plan using this tool. Let us know if you have any questions.

Lastly, could you think of another patient trying to or planning to switch from one SSRI/SNRI to another? Could you briefly describe the patient?

Based on your descriptions, could you make a cross-tapering plan for them using this tool?

Interview questions

1. How did you feel about the overall process?
2. Do you think it aligns well with your current approach of tapering SSRIs and SNRIs?
3. What aspect of the system did you find particularly helpful? Why?
4. What aspect of the system did you find NOT helpful? Why?
5. What kind of care plans that you often administer are well-supported through the tool?
6. What kind of care plans that you often administer are NOT well-supported through the tool?
7. Do you think the system provides enough flexibility to support your regimens of tapering antidepressants? How come?
8. How do you feel about using the tool to create a tapering plan relative to your current approach?
9. How do you feel about using the tool to adjust the tapering plan relative to your current approach?
10. How do you think patients will feel about sharing the information that the tool generated vs. sharing information that you are currently sharing with them?
11. When do you think you are most likely to use this tool? (e.g., before/during/after consultations)
12. Are there any other features that would be useful to be included here?
