# Supplementary material for: Designing Flexible Longitudinal Regimens: Supporting Clinician Planning for Discontinuation of Psychiatric Drugs
Source: Proc SIGCHI Conf Hum Factor Comput Syst. Author manuscript; Available in PMC 2022 Jul 1. (PMC9247721; doi:10.1145/3491102.3502206)
Supplement: Study protocols and term glossary. [file NIHMS1793794-supplement-Study_protocols_and_term_glossary_.zip › Glossary.pdf]

Glossary

Term	Definition
**Cross-taper**	The process of gradually reducing and eventually discontinuing one medication while simultaneously titrating a newly introduced medication to a target dose. An example of a cross-taper involves the gradual switch from an antidepressant with a relatively short half-life (e.g. paroxetine) to one with a long half-life (e.g. fluoxetine) to facilitate antidepressant discontinuation [9].
**Dosage Interval**	In the context of a taper, the duration of time on a certain dosage strength on a drug before reducing to a lower dosage strength. Dosage intervals are typically fixed for a planned taper. Typical dosage intervals when tapering antidepressants are two to four weeks, although in some cases may be longer for certain patients.
**EMR**	Electronic medical records (EMR) refer to computer systems that providers use to create, store, edit, and review patient charts in clinical settings [13]. Common EMR systems used in the U.S. include Epic and Cerner.
**General practitioner**	General practitioners are physicians that treat medical conditions of patients of all ages [16]. The majority of psychiatric care in the United States is provided by general practitioners [5], who may refer patients to psychiatrists if additional expertise is required. The training of a general practitioner consists of undergraduate premedical coursework, four years of medical school to obtain a medical doctorate (MD) or doctorate of osteopathy (DO), and three years of residency at an accredited training site.
**Half-life**	For medical purposes, this refers to a drug's elimination half-life, which is the length of time required for the concentration of a drug in the blood to decrease by 50% [6]. Different drugs have different half-lives. Half-lives of antidepressants generally range from several hours to a few days, although fluoxetine (Prozac®) has an effective half-life of approximately two weeks [14].
**Nurse practitioner**	Nurse practitioners are registered nurses who have undergone postgraduate training [17]. Nurse practitioners are authorized to perform some of the activities of physicians, including prescribing medications, in the United States and increasingly provide frontline psychiatric care.
**Primary care provider**	Providers who provide general care to patients when they first seek medical assistance, perform routine exams, and send referrals to other medical services if patients need specialized treatment. Primary care providers include general practitioners, internists, and nurse practitioner [15].
**Psychiatrist**	Physicians who specialize in mental and behavioral health. In the United States, the training of board-certified psychiatrists consists of undergraduate premedical coursework, four years of medical school to obtain a medical doctorate (MD) or doctorate of osteopathy (DO) four years of residency training in psychiatry, and passage of a board-certification examination [18].
**Scored tablets**	Tablets embossed with a line or multiple lines to facilitate splitting [7].
**SNRI**	Serotonin-norepinephrine reuptake inhibitor. A class of antidepressant drugs that followed SSRIs and possess a dual mechanism of action, although have not been established as more efficacious than SSRIs and may be associated with a broader range of side effects [12]. Examples include venlafaxine (Effexor®) and duloxetine (Cymbalta®).
**SSRI**	Selective-serotonin reuptake inhibitor. The most commonly prescribed class of antidepressant drugs [1]. Examples include escitalopram (Lexapro®) and Fluoxetine (Prozac®).
**Taper**	A gradual reduction in the dose of a drug, typically over the course of several weeks to months in order to avoid undesirable effects that may occur with rapid cessation. [2]

Term	Definition
**Therapeutic and subtherapeutic doses**	A therapeutic dose of an antidepressant refers to a dosage strength that has been established in clinical studies to effectively treat a target condition (e.g. major depressive disorder). Subtherapeutic dose means a dose below the range typically expected to achieve the desired therapeutic effect by the clinical literature [3].
**Withdrawl symptoms**	One or more symptoms that start soon after the discontinuation of a medication, typically within a few days [8]. Examples of withdrawal symptoms associated with antidepressant discontinuation include flu-like symptoms, insomnia, nausea, and sensory disturbances [4, 10, 11, 14].

References

[1] Marwan Alrasheed, Ana L Hincapie, and Jeff J Guo. "Drug Expenditure, Price, and Utilization in the U.S. Medicaid: A Trend Analysis for SSRI and SNRI Antidepressants from 1991 to 2018." In: *The journal of mental health policy and economics* 24.1 (2021), pp. 3–11. ISSN: 1091-4358. URL: http://www.ncbi.nlm.nih.gov/pubmed/33739932.

[2] American Psychiatric Association. *Tapering.* Accessed Sep 6th, 2021. URL: https://dictionary.apa.org/tapering.

[3] Lewis Cooney et al. "Overview of systematic reviews of therapeutic ranges: methodologies and recommendations for practice". In: *BMC Medical Research Methodology* 17.1 (2017). DOI: 10.1186/s12874-017-0363-z.

[4] James Davies and John Read. "A systematic review into the incidence, severity and duration of antidepressant withdrawal effects: Are guidelines evidence-based?" In: *Addictive Behaviors* 97.September 2018 (2019), pp. 111–121. ISSN: 18736327. DOI: 10.1016/j.addbeh.2018.08.027. URL: https://doi.org/10.1016/j.addbeh.2018.08.027.

[5] Bradley N. Gaynes et al. "Major depression symptoms in primary care and psychiatric care settings: A cross-sectional analysis". In: *Annals of Family Medicine* 5.2 (2007), pp. 126–134. ISSN: 15441709. DOI: 10.1370/afm.641.

[6] D J Greenblatt. "Elimination Half-Life of Drugs: Value and Limitations". In: *Annual Review of Medicine* 36.1 (1985), pp. 421–427. ISSN: 0066-4219. DOI: 10.1146/annurev.me.36.020185.002225. URL: http://www.annualreviews.org/doi/10.1146/annurev.me.36.020185.002225.

[7] Emmanuel Reginald Jacques and Paschalis Alexandridis. "Tablet scoring: Current practice, fundamentals, and knowledge gaps". In: *Applied Sciences (Switzerland)* 9.15 (2019). ISSN: 20763417. DOI: 10.3390/app9153066.

[8] Manish K. Jha, A. John Rush, and Madhukar H. Trivedi. "When discontinuing SSRI antidepressants is a challenge: Management tips". In: *American Journal of Psychiatry* 175.12 (2018), pp. 1176–1184. ISSN: 15357228. DOI: 10.1176/appi.ajp.2018.18060692.

[9] Nikki R. Ogle and Shawn R. Akkerman. "Guidance for the discontinuation or switching of antidepressant therapies in adults". In: *Journal of Pharmacy Practice* 26.4 (2013), pp. 389–396. ISSN: 15311937. DOI: 10.1177/0897190012467210.

[10] John Read. "How common and severe are six withdrawal effects from, and addiction to, antidepressants? The experiences of a large international sample of patients". In: *Addictive Behaviors* 102.July 2019 (2020), p. 106157. ISSN: 18736327. DOI: 10.1016/j.addbeh.2019.106157. URL: https://doi.org/10.1016/j.addbeh.2019.106157.

[11] John Read, Claire Cartwright, and Kerry Gibson. "How many of 1829 antidepressant users report withdrawal effects or addiction?" In: *International Journal of Mental Health Nursing* 27.6 (2018), pp. 1805–1815. ISSN: 14470349. DOI: 10.1111/inm.12488.

[12] Daniel Santarsieri and Thomas L. Schwartz. "Antidepressant efficacy and side-effect burden: A quick guide for clinicians". In: *Drugs in Context* 4 (2015), pp. 1–12. ISSN: 17404398. DOI: 10.7573/dic.212290.

[13] Zhiping Walter and Melissa Succi Lopez. "Physician acceptance of information technologies: Role of perceived threat to professional autonomy". In: *Decision Support Systems* 46.1 (2008), pp. 206–215. ISSN: 01679236. DOI: 10.1016/j.dss.2008.06.004. URL: http://dx.doi.org/10.1016/j.dss.2008.06.004.

[14] Christopher H. Warner et al. "Antidepressant discontinuation syndrome". In: *American Family Physician* 74.3 (2006), pp. 449–456. ISSN: 0002838x.

[15] WebMD. *PCP (primary care physician or primary care provider).* Accessed Sep 6th, 2021. URL: https://www.webmd.com/health-insurance/terms/pcp.

[16] WebMD. *What is a general practitioner?* Accessed Sep 6th, 2021. URL: https://www.webmd.com/a-to-z-guides/what-is-a-general-practitioner.

[17] WebMD. *What is a nurse practitioner?* Accessed Sep 6th, 2021. URL: https://www.webmd.com/a-to-z-guides/what-is-a-nurse-practitioner.

[18] WebMD. *What is a psychiatrist?* Accessed Sep 6th, 2021. URL: https://www.webmd.com/a-to-z-guides/what-is-psychiatrist.
