# Supplementary material for: Designing Flexible Longitudinal Regimens: Supporting Clinician Planning for Discontinuation of Psychiatric Drugs
Source: Proc SIGCHI Conf Hum Factor Comput Syst. Author manuscript; Available in PMC 2022 Jul 1. (PMC9247721; doi:10.1145/3491102.3502206)
Supplement: Study protocols and term glossary. [file NIHMS1793794-supplement-Study_protocols_and_term_glossary_.zip › Need-finding study protocol.pdf]

Need-finding study protocol

Introduction

A high-profile BMJ article was recently published, highlighting the need for increased awareness of antidepressant withdrawal and for urgent revisions to antidepressant tapering guidelines, following increased reports of patients with persistent withdrawal symptoms despite standard approaches to tapering antidepressants. NICE guidelines from the UK were subsequently updated in September of 2020 to reflect these concerns.

As a research group, we are working on developing an application to facilitate the provider-patient process of tapering antidepressants, specifically SSRIs and SNRIs, and particularly for those experiencing withdrawal that is refractory to a standard taper protocol.

Demographic Information

Title (e.g., resident psychiatrist PGY-III/PGY-IV, board-certified psychiatrist):
Age:
Gender:
Years of experience post-residency:

Interview questions

Understanding approach to managing tapering

1. In the last 12 months, approximately how many times have you tapered a patient off of an SSRI/SNRI antidepressant?

2. On a scale of 1-10, how comfortable would you be developing a taper for a typical patient? (1 being extremely uncomfortable, 10 being extremely comfortable). [If they don't understand what we mean by typical, imagine they have a patient with major depressive disorder who is stable for 12 months on sertraline 100mg/daily]

3. When configuring a taper, how do (or would you) you calculate dosages? What about this process do you (or would you) find challenging?

4. (probing question) Let's say you have a patient with major depressive disorder who is stable for 12 months on sertraline 100mg/daily. You have decided along with the patient to taper the medication.

   - What would your standard taper approach be?
   - What would you specifically do if the patient developed withdrawal symptoms after a prescription of (insert some aspect of the standard taper approach presented by the provider, preferably after stopping the last dose before full discontinuation) mg?

5. Do you (or would you) ever have to look anything up about the drug or dosage formulations (e.g., capsules, liquid) when trying to design a taper? If so, how do you (or would you) look this information up? What about this process do you (or would you) find challenging?

6. What do you (or would you) do when you have a patient who experiences withdrawal when following your standard taper regimen? What if they're experiencing relapse? What about these processes do you (or would you) find challenging?

Understanding how technology could improve the tapering management process

We are interested in designing technology that could improve the tapering management process. We envision such technology to take the form of an app or/and a website.

1. What kinds of features would you want an app or website for designing tapers to include? Why would those features be helpful?

2. Would you want the app or website to include any data about your patient's:

   - Habits or experiences before or during the taper? Why or why not?

- Medication adherence? Why or why not?
- Aspects of their daily wellbeing? Why or why not?

3. How would you want to use such an app when in consultation with a patient? How would you want to use such an app before or after?
   - Would you want it to support showing the patient a taper schedule? If so, how?
   - Would you want it to support configuring aspects of a taper? If so, how would you want it to support configuring? How?
   - Would you want it to review patients' conditions and medication adherence during the taper? If so, how?
4. Could you benefit from other feedback from patients (anything that's not data that would help understand the patient's experiences)? What kind of feedback?
5. Is there anything else you would like to add?
