# Supplementary material for: Designing Flexible Longitudinal Regimens: Supporting Clinician Planning for Discontinuation of Psychiatric Drugs
Source: Proc SIGCHI Conf Hum Factor Comput Syst. Author manuscript; Available in PMC 2022 Jul 1. (PMC9247721; doi:10.1145/3491102.3502206)
Supplement: Study protocols and term glossary. [file NIHMS1793794-supplement-Study_protocols_and_term_glossary_.zip › Screenshots of a low-fidelity prototype.pdf]

Screenshots of a low-fidelity prototype

We developed a low-fidelity prototype in Mockflow based on the design guidelines derived from the need-finding study.

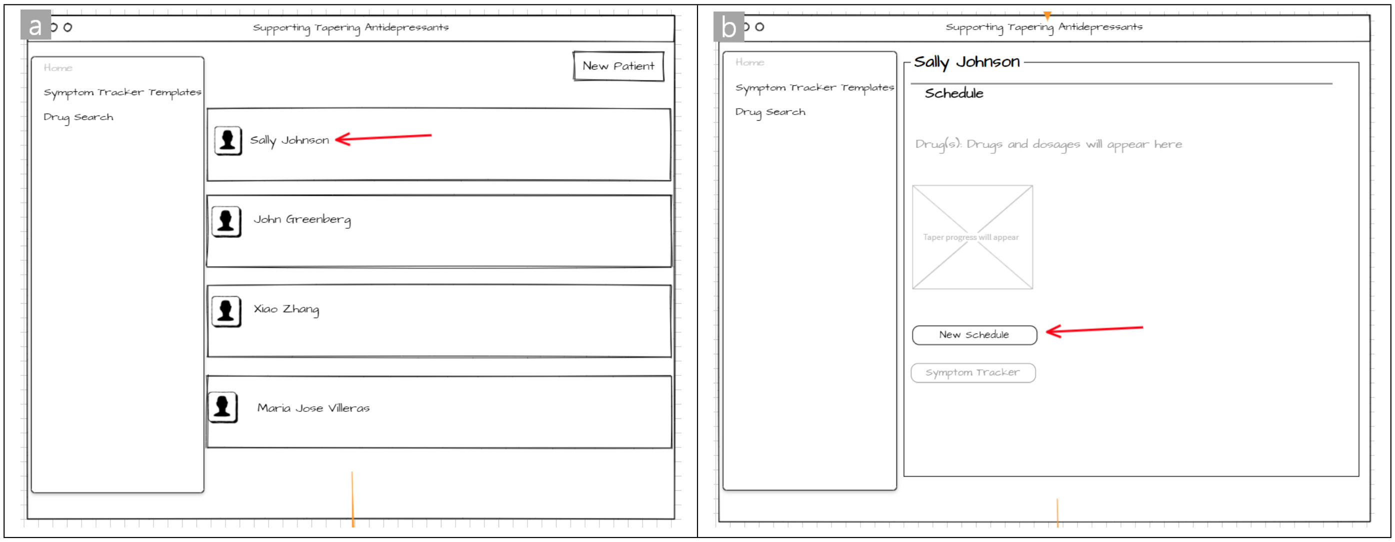

Figure 1: (a) Patients page shows the list of patients visiting the providers. (b) When a patient is selected, information about the patient shows up. Since the selected patient, Sally Johnson, does not have previous visit record, the page renders "New Schedule" button.

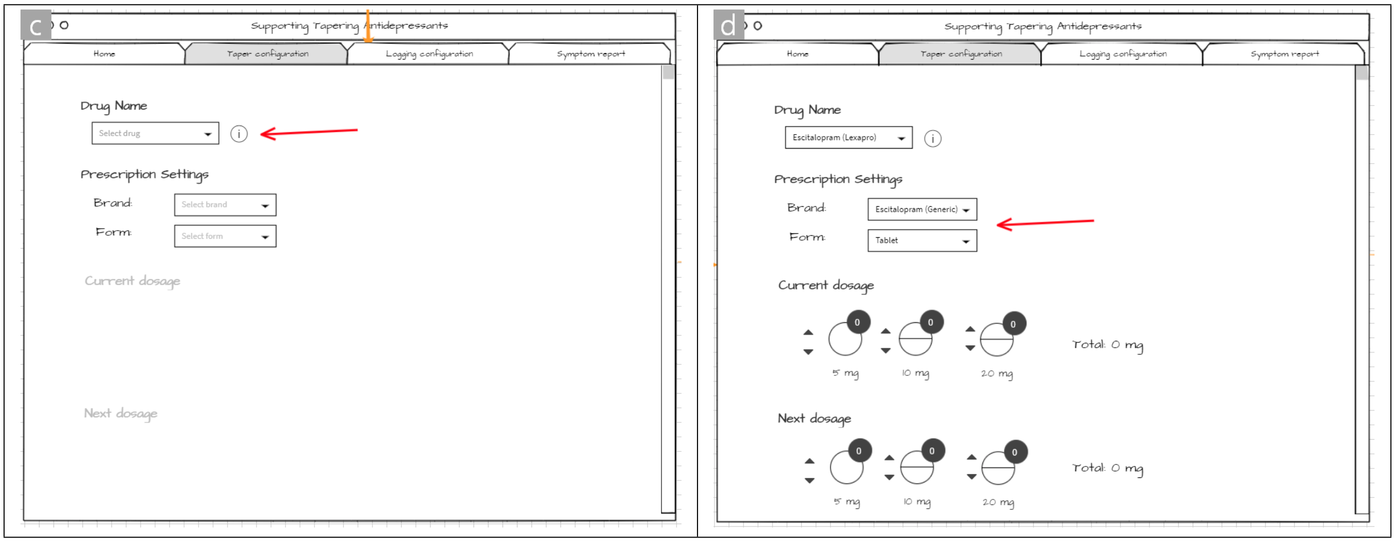

Figure 2: (c) Providers are first asked to choose the name of medication to prescribe. Registered brand and generic options as well as forms for each brand are then given. (d) Providers are then asked to select a brand and a form to prescribe.

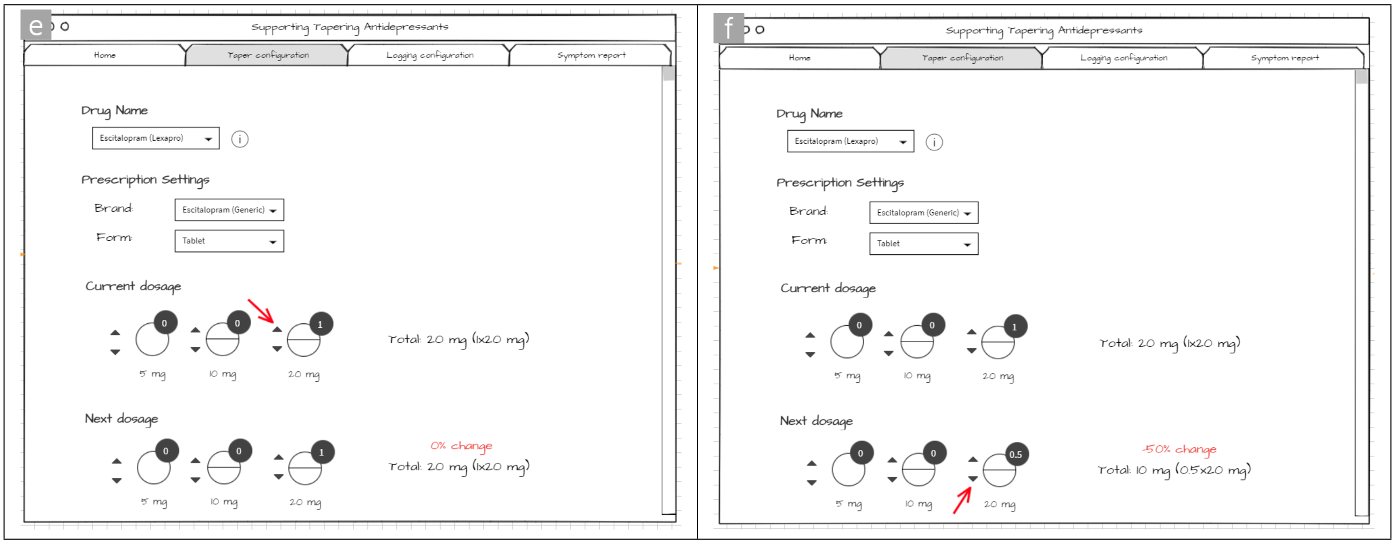

Figure 3: (e) Providers can set the current dosage, the dose a patient is currently on, and (f) the next dosage by clicking on the arrows next to each dose of pill

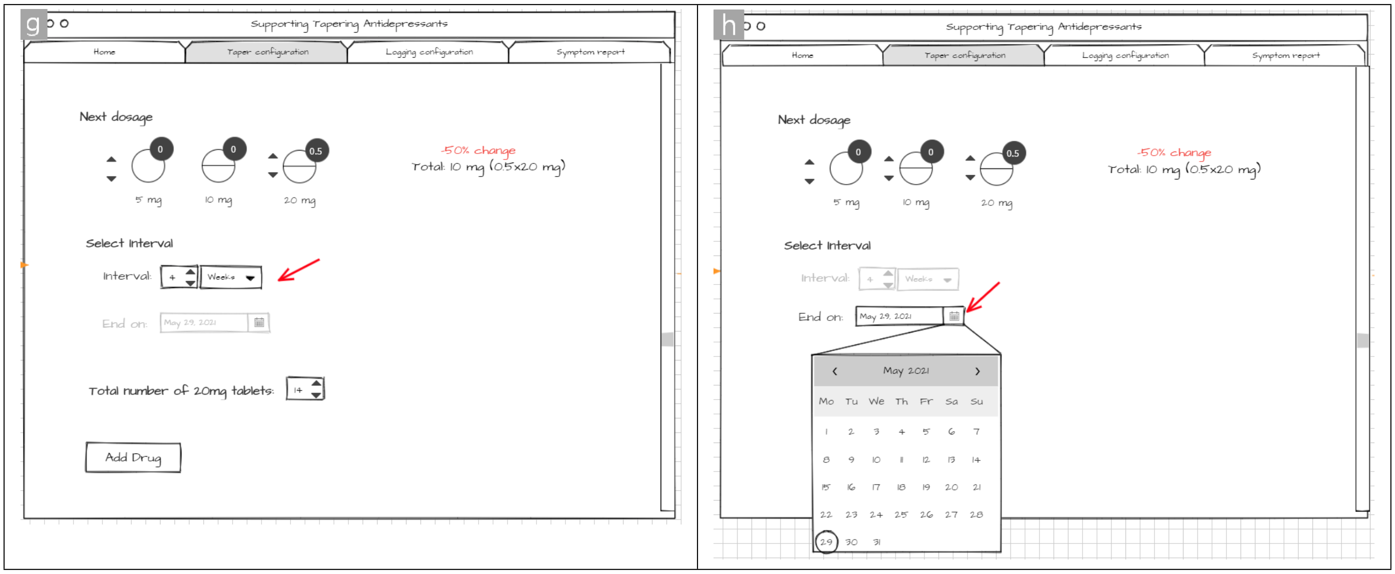

Figure 4: Providers are asked to set the duration of each interval by choosing (g) start date and (h) end date. Providers can also edit the total number of each pill.

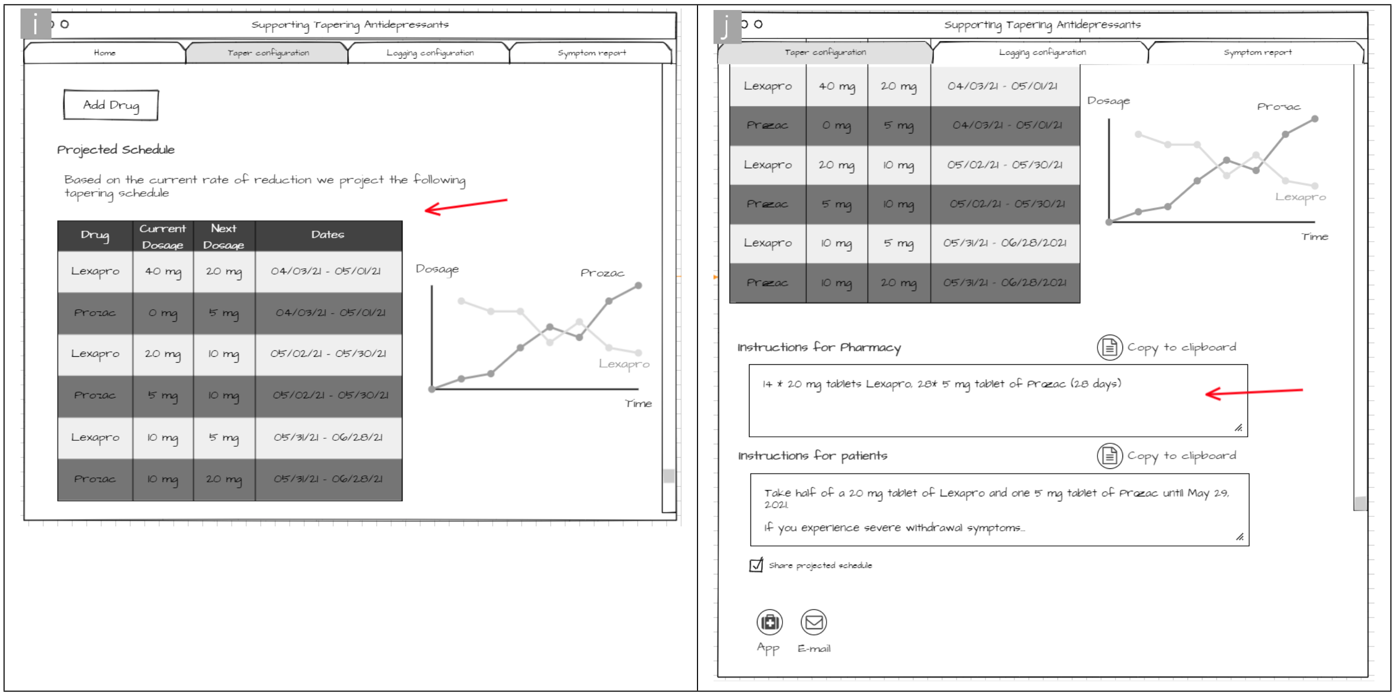

Figure 5: (i) Based on the configured settings, AT Planner projects a potential schedule in a table and a line chart. (j) AT Planner automatically generates the instructions for pharmacy and patients.

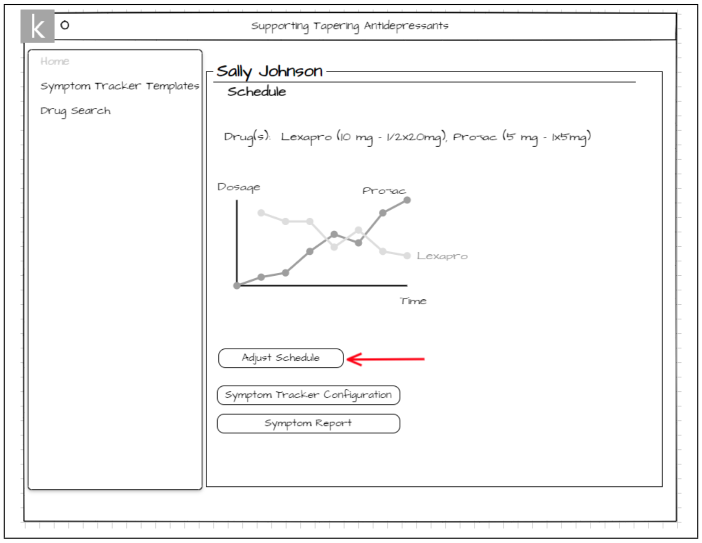

Figure 6: (k) Tapering schedule is saved in a patient page and can be adjusted when needed.
