# Supplementary material for: Designing Flexible Longitudinal Regimens: Supporting Clinician Planning for Discontinuation of Psychiatric Drugs
Source: Proc SIGCHI Conf Hum Factor Comput Syst. Author manuscript; Available in PMC 2022 Jul 1. (PMC9247721; doi:10.1145/3491102.3502206)
Supplement: Study protocols and term glossary. [file NIHMS1793794-supplement-Study_protocols_and_term_glossary_.zip › Screenshots of AT Planner.pdf]

Screenshots of AT Planner

After our verification study with the low-fidelity prototype, we developed a high-fidelity prototype, AT Planner based on the revised design guidelines. AT Planner was developed using React in TypeScript.

Here we describe each screen of AT Planner. The demo video shows the process of interacting with the tool.

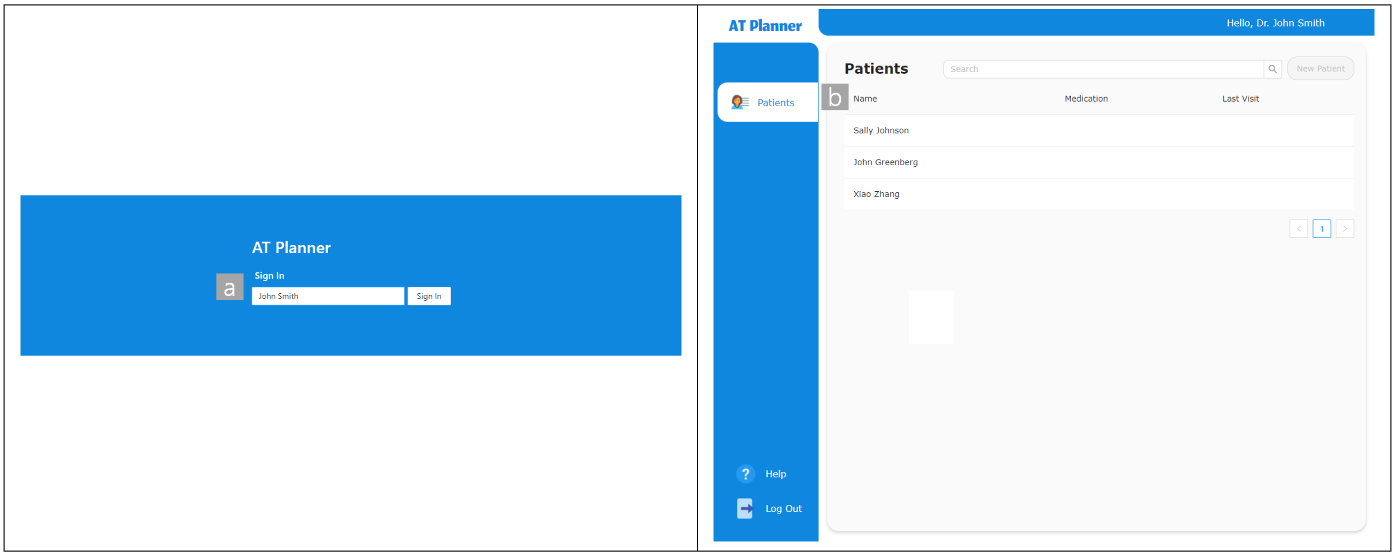

Figure 2: (a) Providers start a session by signing in with their name. (b) After signing in, AT Planner shows a list of patients. Note that all names are pseudonyms and all patients are hypothetical.

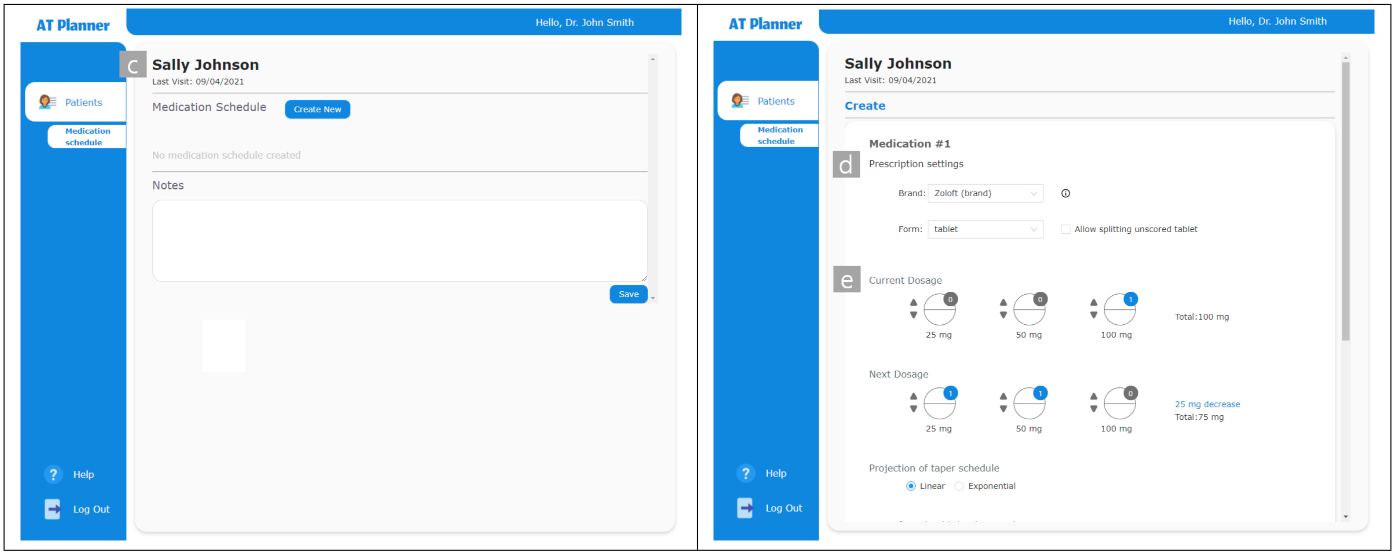

Figure 3: (c) After selecting a patient, providers can create a new tapering plan. (d) Providers are first asked to choose a medication. Providers are then asked to choose a drug form from available forms of each medication. In the case of a tablet, providers are given an option of whether they would allow cutting unscored tablets. (e) Providers set the current dosage which is the dose a patient is currently on, and the next dosage. Providers are given two proejection modes: linear and exponential. Depending on the chosen mode, AT Planner projects the dosage based on the fixed amount or a fixed rate based on the difference between the current and next dosage.

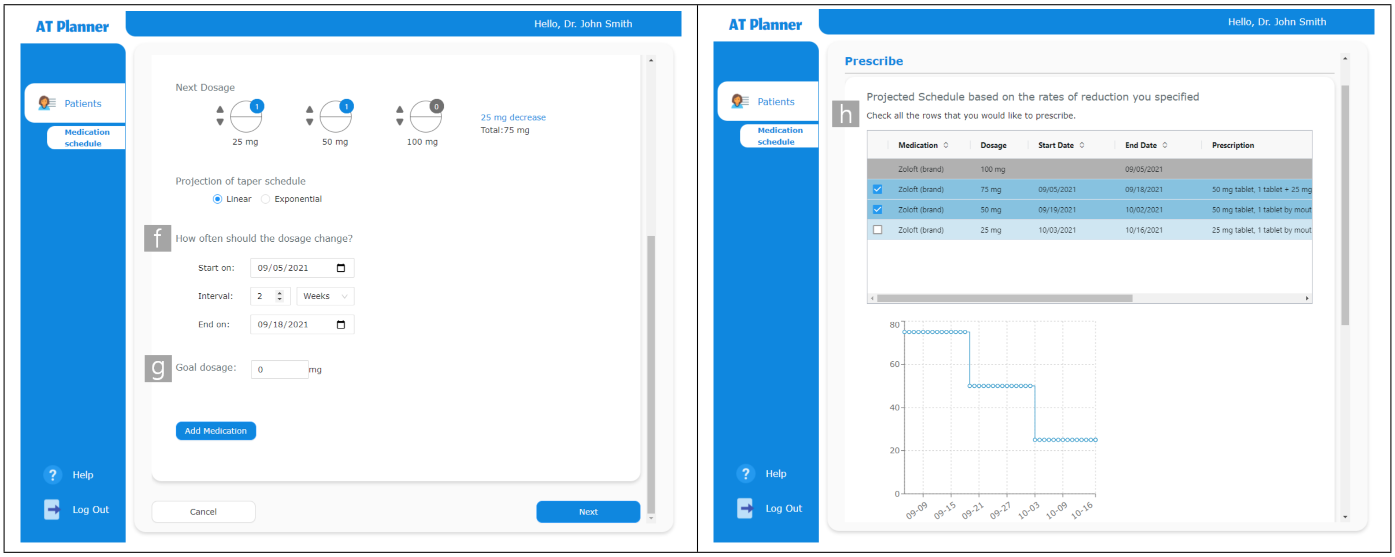

Figure 4: (f) Providers are asked to set the duration of each interval, which is determined by choosing start date and the interval or end date. (g) AT Planner projects dosages until the goal dosage is reached. (h) Based on the configured settings, AT Planner projects a tentative taper schedule in a table and a line chart. Each row in the table represents an interval, and selected intervals are included in the notes for patient and pharmacy (see Figure 5 (i)). The line chart highlights the dosages and reduction rate across the schedule.

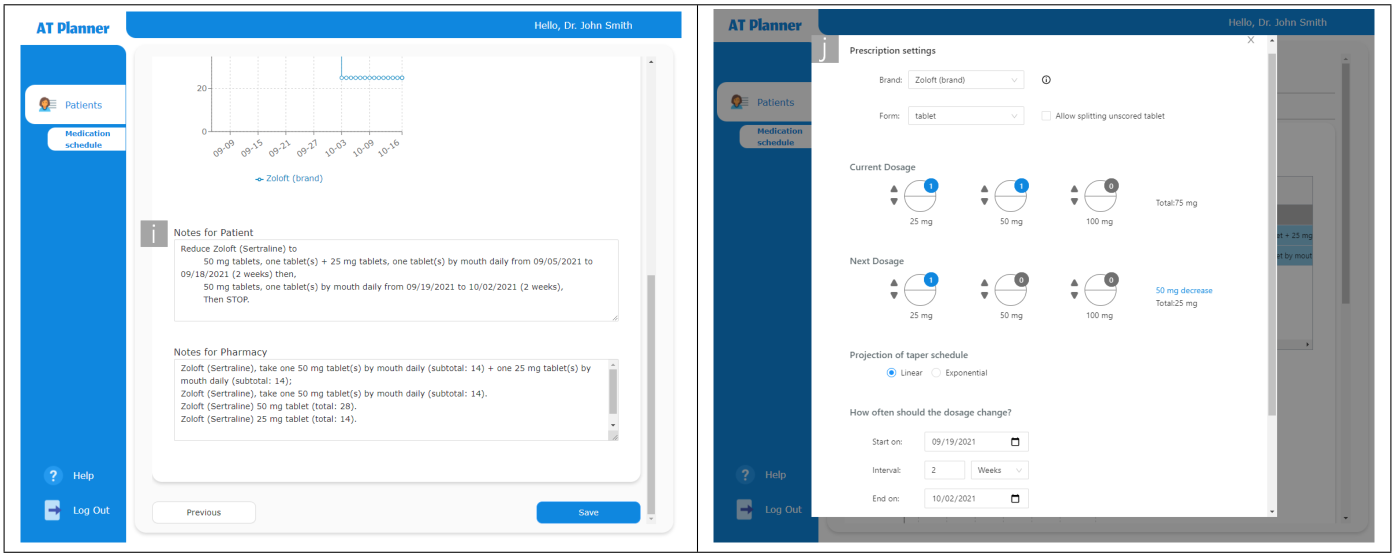

Figure 5: (i) AT Planner automatically generates notes for patient and pharmacy for the selected intervals (see Figure 4 (h)). Providers can also manually edit the notes to add additional instructions. (j) When providers click on a row in the table (see Figure 4 (h)), a modal pops enables providers to edit the drug prescribed, reduction rate, or duration of projected intervals in AT Planner.

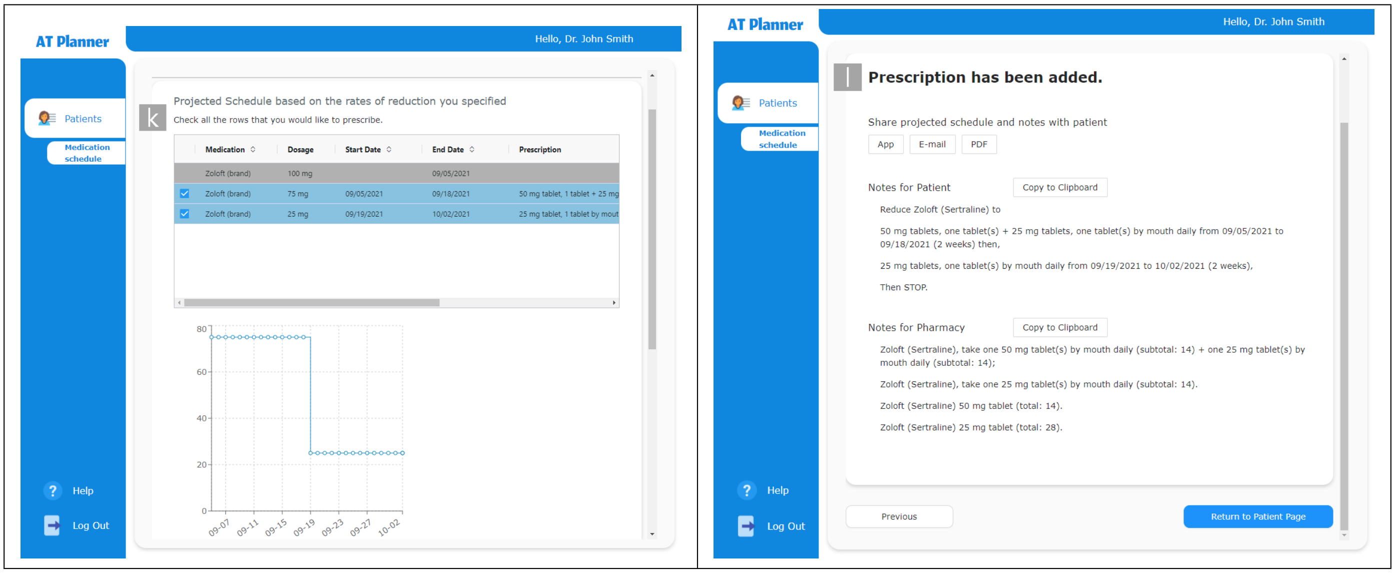

Figure 6: (k) The changes made in the modal are applied to the projected schedule. (l) AT Planner shows the prescriptions generated. This page provides options for sharing the projected schedule with patient and a copy-to-clipboard feature to allow providers to connect the prescriptions to EMR systems or patients' after visit summaries. These features were not functional, but intended to demonstrate how AT Planner could be used in practice.
