# Supplementary material for: Designing Flexible Longitudinal Regimens: Supporting Clinician Planning for Discontinuation of Psychiatric Drugs
Source: Proc SIGCHI Conf Hum Factor Comput Syst. Author manuscript; Available in PMC 2022 Jul 1. (PMC9247721; doi:10.1145/3491102.3502206)
Supplement: Study protocols and term glossary. [file NIHMS1793794-supplement-Study_protocols_and_term_glossary_.zip › Verification study protocol.pdf]

Verification study protocol

Introduction

Thanks so much for your participation in this study. Based on insights from our interview with you and other providers, we developed a prototype of a desktop app that supports taper configuration. Today, we will walk you through a hypothetical scenario. And then ask for your feedback on the usefulness of the design and whether we have implemented what you hoped to get through this tool. Please feel free to interrupt if you have any questions. Our design is still in progress, and we are open to your feedback to improve on it. Any feedback will be appreciated.

Walking through the taper configuration prototype

Let's assume you want to create a new tapering schedule for one of your patients, Sally Johnson. To create a new tapering schedule, you start by searching for the specific drug to taper. Let's assume you selected Lexapro. You are then asked to select the type of prescription, generic or brand. Based on the drug you chose, you can choose one of its available forms, tablet or liquid in this case. Once you make this choice you will see in what forms Lexapro tablets come. In this case 5 mg unscored, 10 mg scored, and 20 mg scored. You are now ready to update the current dosage for Sally's medication. Let's assume she is on 20 mg of Lexapro by taking 1 whole tablet daily. Let's assume you want to decrease Sally's medication from 20 to 10 mg but you want her to use the existing 20 mg pills she has at home. You can specify that she will use half of the 20 mg scored pill. Your final step is to specify the duration of this taper. You can either choose how long the patient should stay on the next dosage. Or you can set the end date in a calendar. Once you complete this step, the tapering configuration is complete. If you want to cross-taper Sally from Lexapro to Prozac, you can add a new drug and repeat the same process. You are then shown a projection of the tapering schedule assuming the same interval and rate of change of Lexapro and Prozac. On the right, we provide a sample graph of the taper. Lastly, the tool creates a text summarizing the prescription to share with the patient or to simply paste into the EHR. The prescription instructions get prepopulated but can be edited to add more specific instructions, e.g., take in the morning or after a meal.

Questions

1. Would these designs be helpful for configuring a taper in your practice?
2. Is there any other data that would be useful to be included here?
3. Are we missing anything?
